# Supplementary material for: Depletion of IL-10 in CAR-NK cells augments reprogramming of the tumor microenvironment and ameliorates therapeutic efficacy
Source: Mol Ther Oncol. 2026 Jun 30;34(3):201285. doi: 10.1016/j.omton.2026.201285 (PMC13382182; doi:10.1016/j.omton.2026.201285)
Supplement: Document S1. Figures S1–S8, Tables S1–S4, and Method S1 [file mmc1.pdf]

OMTON, Volume *34*

Supplemental information

Depletion of IL-10 in CAR-NK cells augments reprogramming of the tumor microenvironment and ameliorates therapeutic efficacy

Anja Löwe, Jasmin Röder, Aline Häcker, Anita Bhatti, Margarete Mijatovic, Nina Müller, Malena Schnieder, Anne Kiefer, Ines Kühnel, Torsten Tonn, Manuel Kaulich, Stefan Stein, Congcong Zhang, Andreas Weigert, and Winfried S. Wels

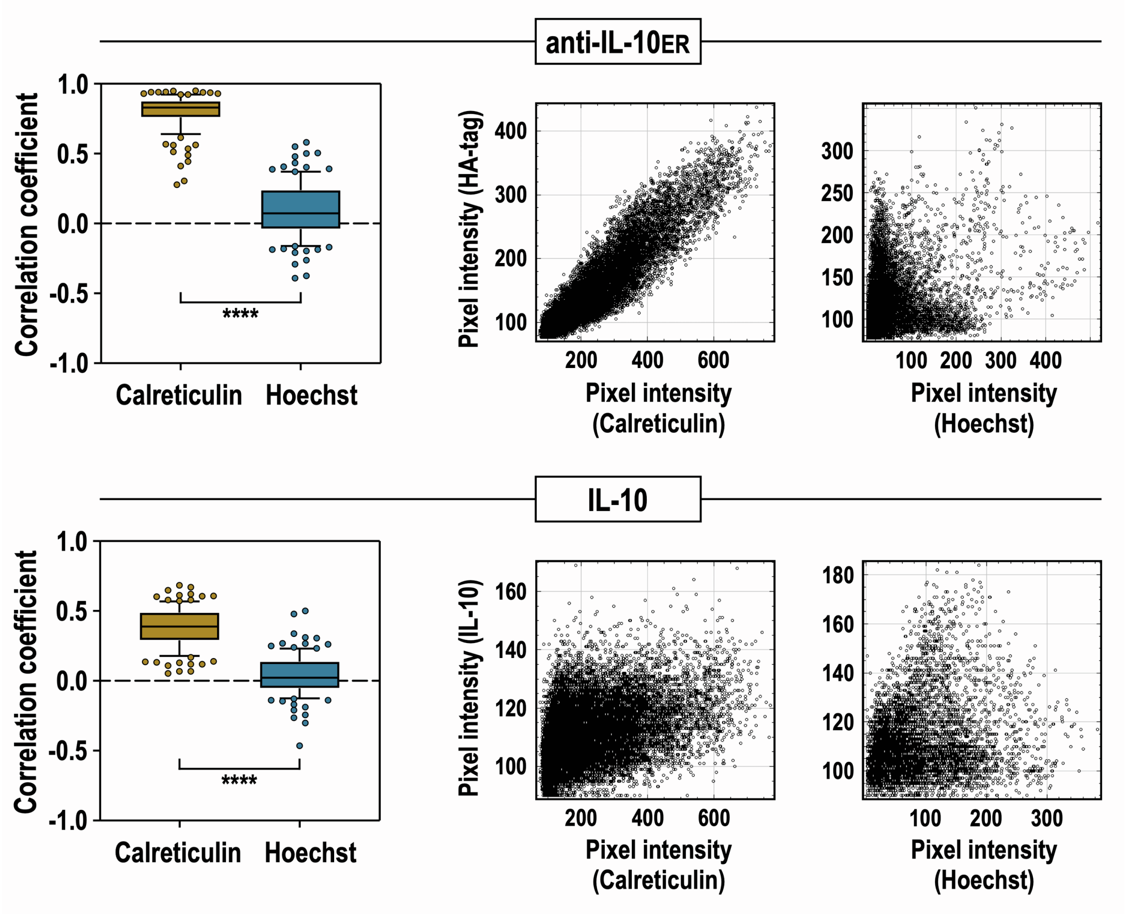

Figure S1. Colocalization of anti-IL-10ER antibody and IL-10 in the endoplasmic reticulum of NK-92/5.28.z/anti-IL10ER cells

NK-92/5.28.z/anti-IL10ER cells were activated by stimulation with PMA and ionomycin, fixed, permeabilized, and stained with antibodies specific for intracellular anti-IL-10ER antibody (HA-tag), calreticulin, and IL-10. Nuclei were stained with Hoechst dye. Images were processed using a customized macro, and colocalization analysis was conducted with the EzColocalization plugin in ImageJ. Left: Colocalization analysis is displayed as Pearson's correlation coefficients (PCC) between anti-IL-10ER and calreticulin or Hoechst (upper panel), or IL-10 and calreticulin or Hoechst (lower panel). PCC values were calculated for individual cells and summarized as whisker box plots representing the 10th–90th percentiles. Data were derived from six images per group. Right: Representative scatter plots, where each pixel's intensity in each channel is plotted, illustrating the degree of colocalization. ****, $p<0.0001$.

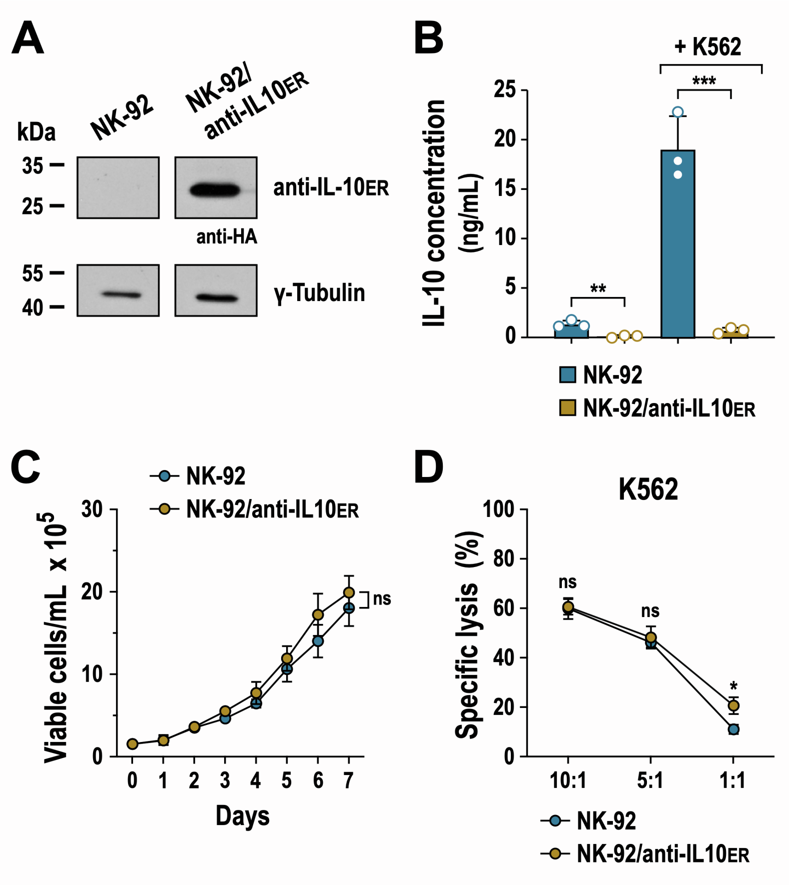

Figure S2. Expression of intracellular anti-IL-10 antibody in parental NK-92 cells
(A) Immunoblot analysis of whole cell lysates of NK-92 and NK-92/anti-IL10ER cells generated by lentiviral transduction of parental NK-92 cells with anti-IL-10ER-encoding vector as described in the main text for CAR-positive NK-92/5.28.z cells. The anti-IL-10ER molecule was detected with HA-tag-specific antibody. γ-tubulin served as a loading control. (B) IL-10 secretion by NK-92 and NK-92/anti-IL10ER cells at steady state and following activation by co-incubation with K562 target cells for 6 hours at an effector to target (E/T) ratio of 1:1 was assessed using a cytometric bead array. Data are shown as mean ± SD from three independent experiments. ***, $p < 0.001$; **, $p < 0.01$. (C) Proliferation of NK-92 and NK-92/anti-IL10ER cells. Cell counts were determined daily for 7 days, with dead cells excluded by trypan blue staining. Data are shown as mean ± SD from three independent experiments. ns (not significant), $p \geq 0.05$. (D) Cytotoxic activity of NK-92 and NK-92/anti-IL10ER cells against K562 erythroleukemia cells was investigated in flow cytometry-based assays after co-incubation with target cells for 2 hours at the indicated effector to target (E/T) ratios. Data are shown as mean ± SD from three independent experiments. *, $p < 0.05$; ns, $p \geq 0.05$.

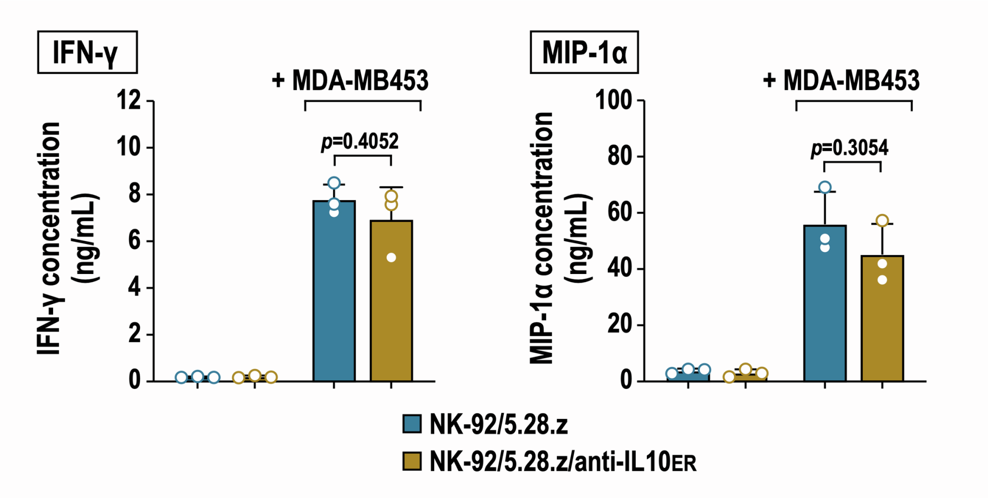

Figure S3. Effects of anti-IL-10ER expression on cytokine production
IFN-γ (left) and MIP-1α secretion (right) by NK-92/5.28.z and NK-92/5.28.z/anti-IL10ER cells at steady state and following CAR activation by co-incubation with ErbB2-positive MDA-MB453 target cells for 6 hours at an effector to target (E/T) ratio of 1:1 was assessed using a cytometric bead array. Data are shown as mean ± SD from three independent experiments.

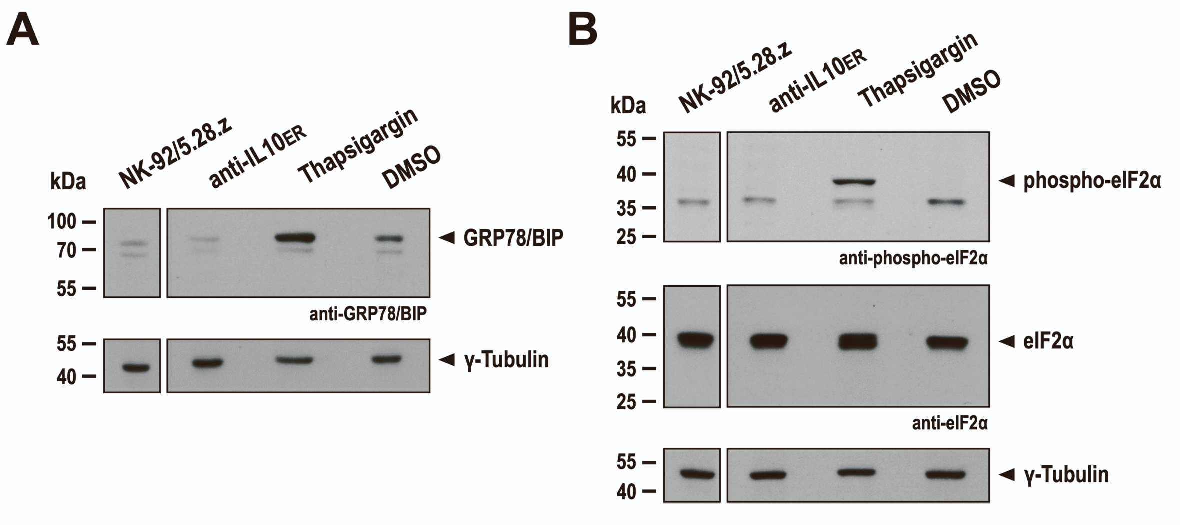

Figure S4. Analysis of ER stress
Whole cell lysates of NK-92/5.28.z and NK-92/5.28.z/anti-IL10ER cells were analyzed by SDS-PAGE and immunoblotting using antibodies specific for the ER chaperone GRP78/BIP (A), or phosphorylated eIF2α (phospho- eIF2α) (B) as markers of ER stress. eIF2α and γ-tubulin served as loading controls. NK-92/5.28.z cells treated with thapsigargin or DMSO were used as controls for ER stress induction.

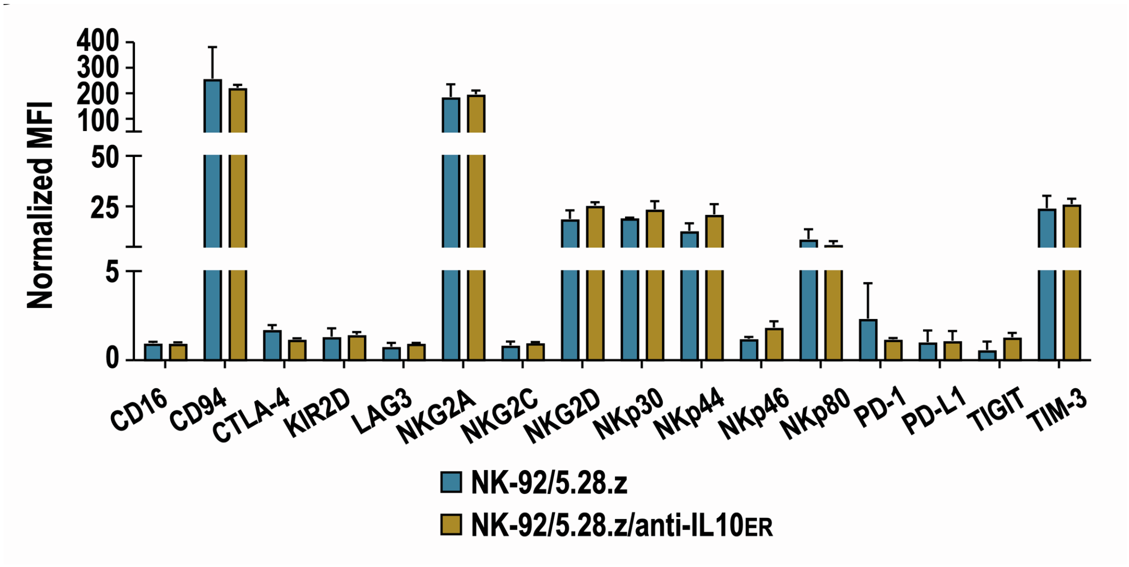

Figure S5. Effects of anti-IL-10ER expression on surface marker expression

Surface expression of the indicated activating and inhibitory NK cell receptors and exhaustion markers on NK-92/5.28.z and NK-92/5.28.z/anti-IL10ER cells was analyzed by staining with specific antibodies and flow cytometry. Mean fluorescence intensities were normalized to unstained controls and are presented as mean values ± SD from three independent experiments.

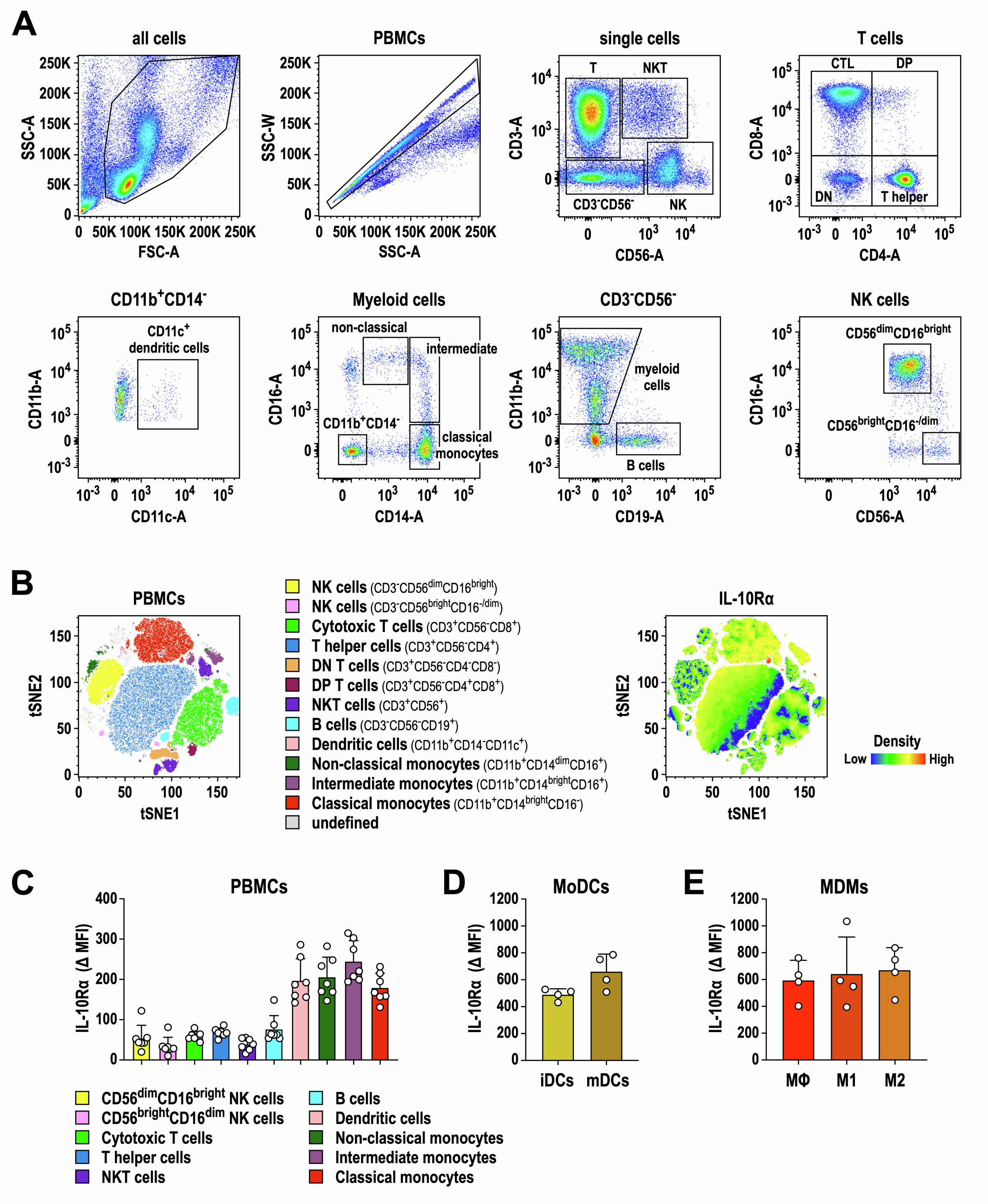

Figure S6. IL-10Rα expression on immune cell subsets

(A) PBMCs from healthy donors were stained with fluorochrome-conjugated antibodies specific for CD3, CD4, CD8, CD11b, CD11c, CD14, CD16, CD19, and CD56 for identification of key immune cell subsets by multicolor flow cytometry. Shown is the gating strategy used to define each subset: T helper cells ($CD3^{+}CD4^{+}CD56^{-}$), cytotoxic T cells (CTL; $CD3^{+}CD8^{+}CD56^{-}$), double-positive T cells (DP; $CD3^{+}CD4^{+}CD8^{+}$), double negative T cells (DN; $CD3^{+}CD4^{-}CD8^{-}$), NK cells

($CD3^{-}CD56^{bright}CD16^{-/dim}$ and $CD3^{-}CD56^{dim}CD16^{+}$), NKT cells ($CD3^{+}CD56^{+}$), B cells ($CD3^{-}$ $CD56^{-}$ $CD19^{+}$), dendritic cells ($CD11b^{+}CD11c^{+}CD14^{-}$), classical monocytes ($CD11b^{+}CD14^{high}CD16^{-}$), intermediate monocytes ($CD11b^{+}CD14^{high}CD16^{+}$), and non-classical monocytes ($CD11b^{+}CD14^{low}CD16^{+}$). (B) PBMC samples from 4 healthy donors were merged to create a t-SNE map using FlowJo software. Left panel: Immune cell subsets, defined based on the gating strategy in (A), and color-coded as indicated. Right panel: IL-10Rα expression on the immune cell subsets, with the indicated color gradient illustrating expression levels. (C) Quantification of IL-10Rα surface expression on different immune cell subsets, displayed as delta mean fluorescence intensities (ΔMFI) relative to IL-10Rα FMO controls. Data are shown as mean ± SD from 7 donors. (D) Surface expression of IL-10Rα on monocyte-derived (MoDC) immature (iDCs) and mature dendritic cells (mDCs). Data are presented as ΔMFI with mean ± SD from 4 donors. (E) Surface expression of IL-10Rα on monocyte-derived macrophage (MDM) subsets, including unpolarized (MΦ), M1-polarized, and M2-polarized macrophages, was quantified as ΔMFI. Data represent mean ± SD from 4 donors.

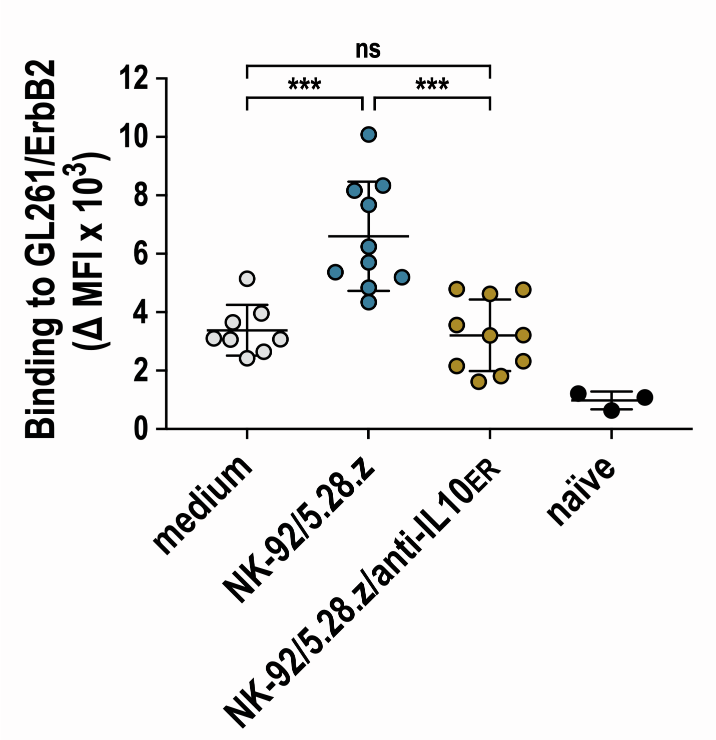

Figure S7. Tumor-specific serum antibody responses in CAR-NK-treated mice
Induction of IgG serum antibodies against GL261/ErbB2 glioblastoma cells in mice treated with injection medium (n=8), NK-92/5.28.z cells (n=10) or NK-92/5.28.z/anti-IL10ER cells (n=10) from the experiment shown in Figure 4D was investigated by flow cytometry with sera collected at the time of sacrifice, and quantified as ΔMFI relative to sera from naïve C57BL/6 mice (n=3). Results are presented as individual data points and means ± SD. ***, $p < 0.001$; ns, $p \geq 0.05$.

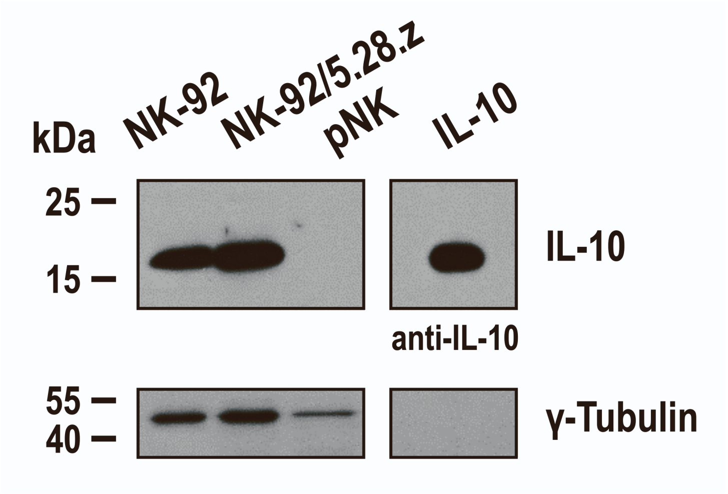

Figure S8. Activation-induced expression of IL-10 in NK cell lines and primary NK cells
Peripheral blood NK (pNK) cells from healthy donors were isolated fom buffy coats and expanded in medium containing 500 IU/mL IL-2 and 50 ng/mL IL-15 as described in Kiefer, A. et al. (2024), Cells 13, 246; doi: 10.3390/cells13030246. Parental NK-92, NK-92/5.28.z CAR-NK and *ex vivo* expanded pNK cells were activated for 5 hours with PMA/ionomycin in the presence of GolgiPlug, cell lysates were prepared and analyzed by SDS-PAGE and immunoblotting with anti-IL-10 antibody. Recombinant IL-10 was used as a positive control. γ-tubulin served as a loading control. Shown are representative data from a single pNK donor.

Supplemental Tables

Table S1. Antibodies used for multispectral imaging

Antibody	Clone	Company	Reference Number
CCR7	ERP23192-57	Abcam	ab253187
CD3ε	SP7	Abcam	ab16669
CD4	D7D2Z	Cell Signaling Technology	25229S
CD8α	D4W2Z	Cell Signaling Technology	98941
CD68	PG-M1	DAKO (Agilent Technologies)	M087601-2
CD163	EPR19518	Abcam	ab182422
CD206	polyclonal	Abcam	ab64693
FoxP3	D6O8R	Cell Signaling Technology	12653
ErbB2 (HER2)	CL0268	Atlas Antibodies	AMAb90838
IL12p35	polyclonal	Thermo Fisher Scientific	PA5-79460

Table S2. Antibodies used for flow-cytometric analysis

Antibody	Clone	Company	Reference Number
CD3	17A2	BioLegend	100220
CD3	UCHT1	BioLegend	100248
CD4	RM4-5	BD Biosciences	553051
CD4	A161A1	BioLegend	357419
CD8α	53-6.7	BioLegend	100734
CD8	SK1	BioLegend	344748
CD11b	M1/70.15.11.5	Miltenyi Biotec	130-113-236
CD11b	M1/70.15.11.5	Miltenyi Biotec	130-113-231
CD11c	B-ly6	BD Biosciences	563130
CD14	HCD14	BioLegend	325604
CD14	MϕP9	BD Biosciences	61384
CD14	MϕP9	BD Biosciences	555398
CD16	3G8	BD Biosciences	563689
CD19	HIB19	BD Biosciences	557921
CD25	3C7	BD Biosciences	558689
CD45	30-F11	BioLegend	103138
CD45	30-F11	BioLegend	103130
CD56	NCAM16.2	BD Biosciences	562751
CD56	5.1H11	BioLegend	362508
CD83	HB15e	BD Biosciences	740802
CD86	2331	BD Biosciences	555660
CD94	REA113	Miltenyi Biotec	130-123-884
CD163	GHI/61	BD Biosciences	563697
CD206	MR5D3	BD Biosciences	565250

CD210a (IL-10R)	3F9	BioLegend	308812
CD210α (IL-10R)	3F9	BD Biosciences	556013
CTLA-4	L3D10	BioLegend	349908
ErbB2 (HER2)	24D2	BioLegend	324412
F4/80	BM8	BioLegend	123110
FoxP3	MF-14	BioLegend	126419
HA-tag	16B12	BioLegend	682404
HLA-DR	G46-6	BD Biosciences	563083
IL-10	JES3-9D7	BioLegend	501404
KIR2D	NKVFS1	Miltenyi Biotec	130-123-710
LAG-3	7H2C65	BioLegend	369211
Ly6C	HK1.4	BioLegend	128032
Ly6G	1A8	BioLegend	127654
NKG2A	REA110	Miltenyi Biotec	130-113-563
NKG2C	REA205	Miltenyi Biotec	130-117-398
NKG2D	BAT221	Miltenyi Biotec	130-117-718
NKp30	REA823	Miltenyi Biotec	130-112-430
NKp44	p44-8	BD Biosciences	558564
NKp46	9 E2	Miltenyi Biotec	130-128-826
NKp80	4A4.D10	Miltenyi Biotec	130-126-102
PD-1	EH12.2H7	BioLegend	329922
PD-L1	29E.2A3	BioLegend	329718
TIGIT	MBSA43	Invitrogen	12-9500-42
TIM-3	F38-2E2	BioLegend	345026

Table S3. Antibodies used for immunofluorescence microscopy

Antibody	Clone	Company	Reference Number
HA-tag	16B12	BioLegend	682404
IL-10	JES3-9D7	BioLegend	506812
Calreticulin	EPR3924	Abcam	ab92516
CD45	2D1	Invitrogen	17-0459-42
phospho-STAT3	D3A7	Cell Signaling Technology	9145T

Table S4. Antibodies used for immunoblot analysis

Antibody	Clone	Company	Reference Number
HA-tag	C29F4	Cell Signaling Technology	11846S
γ-tubulin	polyclonal	Sigma-Aldrich	T3559
GRP78/BIP	C50B12	Cell Signaling Technology	3177S
phospho-eIF2α	D9G8	Cell Signaling Technology	3398S
eIF2α	D7D3	Cell Signaling Technology	5324S

Supplemental Methods

ImageJ Macro to quantify mean fluorescent intensity of pSTAT3 staining

Language: ImageJ Macro

```
// Set scale in microns
run("Set Scale...", "distance=84.0 known=10 unit=µm");

// Split the channels of the composite image
selectImage("1.tif");
run("Split Channels");
selectImage("C1-1.tif");
rename("Hoechst");
run("Cyan");
run("8-bit");
selectImage("C2-1.tif");
rename("Phospho-Stat3");
run("Yellow");
run("8-bit");
selectImage("C3-1.tif");
rename("CD45");
run("Magenta");
run("8-bit");

// Adjust brightness and contrast
selectImage("CD45");
run("Enhance Contrast", "saturated=0.35");
selectImage("Phospho-Stat3");
setMinAndMax(5, 117);
selectImage("Hoechst");
setMinAndMax(13, 140);

//Create a binary mask for CD45
selectImage("CD45");
setAutoThreshold("Triangle dark no-reset");
//setThreshold(7, 255);
run("Convert to Mask");
run("Close-");
run("Fill Holes");
run("Watershed");
run("Convert to Mask");

//AnalyzeParticles
run("Analyze Particles...", "size=10-Infinity circularity=0.5-1.00 display exclude clear overlay add
composite");
selectImage("Phospho-Stat3");
roiManager("Measure");
```

Image-based analysis of phospho-STAT3 expression in CD45-positive cells. The macro processes multichannel fluorescence images containing Hoechst, phospho-STAT3 and CD45 channels. Images are split into individual channels, converted to 8-bit, and contrast-adjusted. The CD45 channel is thresholded and refined with morphological operations to create a mask of CD45-

positive cells. Particle analysis identifies these cells, generating ROIs, which are then applied to the phospho-STAT3 channel to quantify mean fluorescence intensity (MFI) within CD45-positive cells.
