# Supplementary material for: Depletion of IL-10 in CAR-NK cells augments reprogramming of the tumor microenvironment and ameliorates therapeutic efficacy
Source: Mol Ther Oncol. 2026 Jun 30;34(3):201285. doi: 10.1016/j.omton.2026.201285 (PMC13382182; doi:10.1016/j.omton.2026.201285)
Supplement: Document S2. Article plus supplemental information [file mmc2.pdf]

Molecular Therapy
Oncology
Original Article

Depletion of IL-10 in CAR-NK cells augments reprogramming of the tumor microenvironment and ameliorates therapeutic efficacy

Anja Löwe,[1,8] Jasmin Röder,[1,8] Aline Häcker,[1] Anita Bhatti,[1] Margarete Mijatovic,[2] Nina Müller,[1] Malena Schnieder,[1] Anne Kiefer,[1] Ines Kühnel,[1] Torsten Tonn,[3,4,5] Manuel Kaulich,[4,6] Stefan Stein,[1,4] Congcong Zhang,[1] Andreas Weigert,[2,4,7] and Winfried S. Wels[1,4,5]

[1]Georg-Speyer-Haus, Institute for Tumor Biology and Experimental Therapy, 60596 Frankfurt, Germany; [2]Institute of Biochemistry I, Faculty of Medicine, Goethe University, 60590 Frankfurt, Germany; [3]Institute for Transfusion Medicine and Immunohematology, Goethe University, 60528 Frankfurt and Red Cross Blood Donation Service Baden-Württemberg-Hessen, 60528 Frankfurt, Germany; [4]Frankfurt Cancer Institute, Goethe University, 60590 Frankfurt, Germany; [5]German Cancer Consortium (DKTK), Partner Site Frankfurt/Mainz, 60590 Frankfurt, Germany; [6]Institute of Biochemistry II, Faculty of Medicine, Goethe University, 60590 Frankfurt, Germany; [7]Department for Immunity of Inflammation, Mannheim Institute for Innate Immunoscience, Medical Faculty Mannheim, Heidelberg University, 68167 Mannheim, Germany

The ErbB2 (HER2)-specific CAR-engineered natural killer (NK) cell line NK-92/5.28.z is under investigation in a phase I clinical trial in glioblastoma patients. In preclinical studies, these cells demonstrated potent CAR-mediated cytotoxicity and stimulated endogenous antitumor immunity in immunocompetent animals. Pro-inflammatory cytokines can contribute to the CAR-NK cells' immunomodulatory activity, but this may be attenuated by immunosuppressive IL-10 that is also produced in substantial amounts by activated NK-92/5.28.z cells. To prevent IL-10 secretion, we modified the CAR-NK cells to express an intracellular anti-IL-10 antibody, which trapped IL-10 within the endoplasmic reticulum. This did not affect proliferation, phenotype, or cytotoxicity of the resulting NK-92/5.28.z/anti-IL10ER cells but strengthened their ability to mediate maturation of co-cultured dendritic cells and prevented M2-polarization of co-cultured macrophages induced by unmodified CAR-NK cells. In a syngeneic murine glioblastoma model, NK-92/5.28.z/anti-IL10ER cells exhibited enhanced antitumor activity and favored a pro-inflammatory tumor microenvironment characterized by reduced infiltration of IL-10-responsive immunosuppressive cell types. Our findings demonstrate that inhibiting IL-10 secretion improves the therapeutic potential of CAR-engineered NK-92 cells, suggesting this approach as a promising avenue for clinical translation.

INTRODUCTION

Adoptive immunotherapy with chimeric antigen receptor (CAR)-engineered T cells has demonstrated impressive efficacy in patients with B cell malignancies and has become standard-of-care for these indications.[1] To prevent graft-versus-host disease (GvHD), currently approved CAR-T cell therapies rely on genetic modification of patient-derived, autologous T lymphocytes, which may be functionally compromised due to the underlying disease or prior treatments. In contrast, natural killer (NK) cells and their genetically engineered derivatives can be safely applied even in an MHC-unmatched allogeneic setting due to their lack of a T cell receptor.[2–4] This is underscored by data from a phase I/II clinical trial with cord blood-derived NK cells expressing a CD19-specific CAR, which demonstrated similar activity against B-cell leukemias and lymphomas as CAR-T cells but without inducing GvHD or adverse events such as cytokine release syndrome (CRS) and effector cell associated neurotoxicity syndrome (ICANS) that are common complications of CAR-T cell therapy.[5,6] Likewise, CD19-CAR NK cells derived from induced pluripotent stem cells proved safe and efficacious against B-cell lymphoma in a recent phase I clinical trial.[7] Similar to such donor-derived and *ex vivo* differentiated NK cells, the stable and continuously expanding NK cell line NK-92 allows the generation of cost-effective, CAR-engineered off-the-shelf therapeutics suitable for evaluation in early phase clinical trials.[8–10]

Despite these advances in hematological malignancies, efforts to apply CAR-expressing lymphocytes for the treatment of more prevalent solid cancers have so far demonstrated only limited success, with an immunosuppressive tumor microenvironment, restricted persistence of infused effector cells, and heterogeneity of target antigen expression constituting major hurdles.[11] In this regard, CAR-NK cells may offer advantages, as they retain natural cytotoxicity mediated by recognition of stress-induced ligands on tumor cells via endogenous natural cytotoxicity receptors (NCRs) and NKG2D

Received 9 April 2026; accepted 29 June 2026;
https://doi.org/10.1016/j.omton.2026.201285.

[8]These authors contributed equally

Correspondence: Winfried S. Wels, Georg-Speyer-Haus, Institute for Tumor Biology and Experimental Therapy, 60596 Frankfurt, Germany.
E-mail: wels@gsh.uni-frankfurt.de

Published by Elsevier Inc. on behalf of The American Society of Gene and Cell Therapy.
This is an open access article under the CC BY license (http://creativecommons.org/licenses/by/4.0/).

independent from CAR activation.[4,12] This intrinsic cytotoxicity may render CAR-NK cells less prone to treatment-induced selection of antigen-loss tumor cell variants. An important aspect of the natural antitumor activity of NK cells is also their crosstalk with other immune cell types, in particular, their contribution to the activation of dendritic cells (DCs),[13,14] which is decisive for an effective T cell immune response. Indeed, treatment-induced endogenous antitumor immunity was critical for long-lasting tumor control and cures after therapy with the clonal CAR-engineered NK-92 derivative NK-92/5.28.z in immunocompetent mouse glioma models,[15–18] with signs of treatment-induced immune activation in the tumor microenvironment also observed in glioblastoma patients in the ongoing CAR2BRAIN phase I clinical trial (NCT03383978).[9]

NK-92/5.28.z cells carry a second-generation CAR with a composite CD28 and CD3ζ signaling domain, which specifically recognizes the tumor-associated ErbB2 (HER2) antigen and facilitates targeted and effective cytotoxicity of the CAR-NK cells against ErbB2-positive malignancies, such as breast carcinoma and glioblastoma.[15,19,20] Thereby, activation of NK-92/5.28.z cells not only initiates lysis of cognate targets but also promotes the secretion of pro-inflammatory cytokines like interferon (IFN)-γ and tumor necrosis factor (TNF)-α,[15,19,21] which likely contribute to the immunostimulatory activity of the CAR-NK cells in immunocompetent animal models and cancer patients. Nevertheless, activation of the CAR-engineered NK-92 cells also triggers secretion of large amounts of interleukin (IL)-10,[19,21] a cytokine with well-established anti-inflammatory and immunosuppressive functions. Typically, IL-10 production characterizes a distinct NK cell subset associated with tolerance induction in the placenta during pregnancy.[22] Furthermore, IL-10 produced by NK cells can restrain IL-12 production by DCs, thereby limiting immunopathology during systemic infections.[23] While the contribution of IL-10 produced by NK cells to the immunosuppressive tumor microenvironment is presently unknown, elevated frequencies of IL-10-expressing peripheral blood NK cells have been found in cancer patients.[24] Hence, it is conceivable that IL-10 produced at high levels by activated NK-92/5.28.z cells may, to some extent, counteract the beneficial effects of pro-inflammatory factors like IFN-γ and TNF-α, thereby limiting the therapeutic potential of the CAR-NK cells.

Here, to test this hypothesis and investigate the consequences of preventing IL-10 secretion by NK-92/5.28.z cells, we further engineered the clinical-grade CAR-NK cells to express an intracellular antibody (commonly termed intrabody) specific for IL-10 as an endoplasmic reticulum (ER)-retained molecule, intended to trap the cytokine within the cells. IL-10 production, proliferative potential, and cytotoxicity of the resulting NK-92/5.28.z/anti-IL10ER cells were compared with those of unmodified CAR-NK cells, and their effects on maturation of DCs and polarization of macrophages were analyzed in co-culture experiments *in vitro*. Modulation of the tumor microenvironment by the IL-10-depleted CAR-NK cells and *in vivo* antitumor activity were then evaluated in a syngeneic model of ErbB2-positive glioblastoma in immunocompetent C57BL/6 mice.

RESULTS

Prevention of IL-10 secretion by expression of an ER-retained anti-IL-10 antibody

NK-92/5.28.z cells are a good manufacturing practice (GMP)-compliant, permanently gene-modified single-cell clone of the NK-92 cell line that carries a CAR specific for the tumor-associated ErbB2 (HER2) antigen,[19] consisting of an ErbB2-specific single-chain fragment variable (scFv) antibody fragment, linked via a CD8α hinge region to transmembrane and intracellular domains of CD28 and a CD3ζ signaling domain (Figure 1A). Activation of these CAR-NK cells triggers cytotoxicity against ErbB2-positive targets and secretion of pro-inflammatory cytokines such as IFN-γ and TNF-α,[15,19,21] but it also upregulates production of immunoregulatory IL-10 at both, the transcriptional and the protein level (Figures 1B and 1C). This IL-10 response occurred upon unspecific stimulation with phorbol 12-myristate 13-acetate (PMA) and ionomycin, and to a lesser extent after activation by K562 cells that trigger natural cytotoxicity. In the case of NK-92/5.28.z cells, marked IL-10 production was also induced upon CAR activation by ErbB2-expressing targets such as MDA-MB453 breast carcinoma cells that are resistant to parental NK-92 cells, while no IL-10 secretion was observed upon contact with ErbB2-negative MDA-MB468 breast cancer cells (Figure 1C).

To inhibit IL-10 secretion, we transduced the CAR-NK cells with a lentiviral vector that encodes an IL-10-specific intracellular scFv antibody (anti-IL-10ER), which is directed into the secretory pathway by an N-terminal immunoglobulin heavy chain signal peptide, and carries a C-terminal KDEL peptide sequence for retention in the ER during transit via recognition by the KDEL receptor (Figure 1D).[25,26] Transduced CAR-NK cells were enriched by flow-cytometric cell sorting based on the expression of enhanced green fluorescent protein (EGFP) included as a reporter gene in the anti-IL-10ER-encoding vector. Expression of the antibody fragment by the resulting NK-92/5.28.z/anti-IL10ER cells and its intracellular localization were then confirmed by intracellular staining with an antibody detecting the hemagglutinin (HA)-tag contained in the molecule (Figure 1E) and immunoblot analysis of cell lysates (Figure 1F).

Confocal microscopy further demonstrated predominant localization of the anti-IL-10ER intrabody and IL-10 in the ER with substantial colocalization observed with the ER-resident protein calreticulin (Figure 1G), which was confirmed by quantitative analysis revealing high Pearson's correlation coefficients (PCCs) of 0.8 ± 0.13 for anti-IL-10ER/calreticulin and 0.47 ± 0.34 for IL-10/calreticulin (Figure S1). To further evaluate functionality of the IL-10-specific intrabody, a cytometric bead array (CBA) was used to quantify IL-10 secretion. Following CAR activation with ErbB2-positive MDA-MB453 cells, unmodified NK-92/5.28.z cells released high amounts of IL-10, whereas for activated NK-92/5.28.z/anti-IL10ER cells, IL-10 secretion was reduced to baseline levels of unstimulated NK-92/5.28.z cells (Figure 1H, left). Similarly, parental NK-92 cells produced high levels of IL-10 if activated by exposure to K562

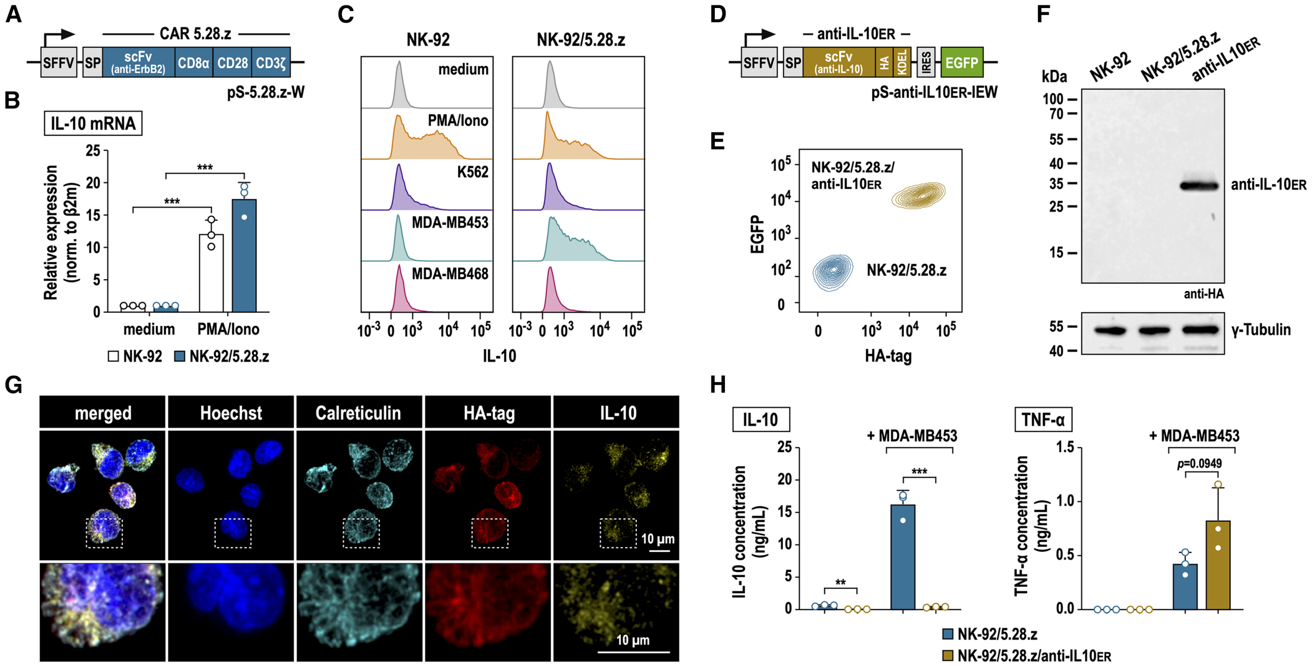

Figure 1. Retention of interleukin-10 in CAR-NK cells by expression of an intracellular anti-IL-10 antibody

(A) ErbB2-specific CAR expressed by NK-92/5.28.z cells. The cells stably express under control of the spleen focus forming virus promoter (SFFV) a CAR introduced by lentiviral transduction, which consists of an immunoglobulin heavy chain signal peptide (SP), an ErbB2-specific antibody fragment (scFv), a modified CD8α hinge region, followed by transmembrane and intracellular domains of CD28, and the intracellular domain of CD3ζ. (B) Activation-induced expression of IL-10 mRNA. NK-92/5.28.z cells were stimulated with PMA and ionomycin for 1 h. Total RNA was isolated, reverse-transcribed to cDNA, and analyzed for IL-10 expression by quantitative real-time PCR. Parental NK-92 cells were included for comparison. IL-10 mRNA expression was calculated relative to baseline values of medium controls. β_2 microglobulin (β2m) served as a reference gene. Data are shown as mean ± SD from three independent experiments. $***p < 0.001$. (C) Activation-induced expression of IL-10 protein. Parental NK-92 and CAR-engineered NK-92/5.28.z cells were incubated for 3 h in the presence of GolgiStop with either PMA and ionomycin, NK-sensitive K562 erythroleukemia cells, ErbB2-positive MDA-MB453 or ErbB2-negative MDA-MB468 breast carcinoma cells. Unstimulated NK cells served as control (medium). IL-10 expression in CD56-positive cells was analyzed by intracellular cytokine staining and flow cytometry. (D) Lentiviral transfer plasmid encoding under control of the SFFV promoter, the intracellular antibody anti-IL-10ER, which consists of an immunoglobulin heavy chain SP, an IL-10-specific antibody fragment (scFv) fused to a hemagglutinin tag (HA) and a KDEL endoplasmic reticulum retention sequence, followed by an internal ribosome entry site (IRES) and enhanced green fluorescent protein (EGFP) as a marker. (E) Flow-cytometric analysis of NK-92/5.28.z/anti-IL10ER cells. Lentivirally transduced cells were identified as EGFP-positive and analyzed for expression of the anti-IL-10ER antibody by staining with HA-tag-specific antibody. NK-92/5.28.z cells served as control. (F) Immunoblot analysis of whole cell lysates of NK-92, NK-92/5.28.z, and NK-92/5.28.z/anti-IL10ER cells (indicated as anti-IL10ER). The anti-IL-10ER molecule was detected with HA-tag-specific antibody. γ-tubulin served as a loading control. (G) Immunofluorescence microscopy of NK-92/5.28.z/anti-IL10ER cells. The NK cells were activated by stimulation with PMA and ionomycin, fixed, permeabilized, and stained with antibodies specific for anti-IL-10ER protein (HA-tag; red), calreticulin as endoplasmic reticulum marker (cyan), and IL-10 (yellow). Nuclei were visualized with Hoechst dye (blue). Representative images are shown. Scale bars, 10 μm. (H) IL-10 (left) and TNF-α secretion (right) by NK-92/5.28.z and NK-92/5.28.z/anti-IL10ER cells at steady state and following CAR activation by co-incubation with ErbB2-positive MDA-MB453 target cells for 6 h at an effector to target (E/T) ratio of 1:1 was assessed using a cytometric bead array. Data are shown as mean ± SD from three independent experiments. $**p < 0.01$; $***p < 0.001$.

erythroleukemia cells, which was prevented upon expression of the IL-10-specific intrabody (Figures S2A and S2B). Interestingly, IL-10-depleted NK-92/5.28.z/anti-IL10ER CAR-NK cells trended to produce more TNF-α upon CAR activation than did NK-92/5.28.z cells ($p = 0.0949$; Figure 1H, right), while IFN-γ and MIP-1α levels were not affected when IL-10 secretion was blocked (Figure S3).

These results confirm expression and intracellular retention of the anti-IL-10ER antibody together with IL-10, and demonstrate its high efficiency in preventing secretion of the cytokine by NK-92/5.28.z/anti-IL10ER CAR-NK cells upon activation by target cells.

Viability and effector functions of IL-10-depleted CAR-NK cells

IL-10 influences activation, cytokine secretion, and cytotoxicity of primary NK cells.[27] Established NK-92 cells and their CAR-expressing derivatives endogenously express the IL-10 receptor (Figure 2A) and produce low basal levels of IL-10 even in the absence of target cells (see Figure 1H, left), which may act on the cells in an autocrine manner. To evaluate the consequences of trapping IL-10 within NK-92/5.28.z/anti-IL10ER cells on signaling events downstream of the IL-10 receptor, we examined phosphorylation of signal transducer and activator of transcription (STAT) 3 following IL-2 stimulation, IL-2 starvation, or IL-2 starvation with subsequent activation by PMA and ionomycin (Figures 2B and 2C). Quantitative analysis of

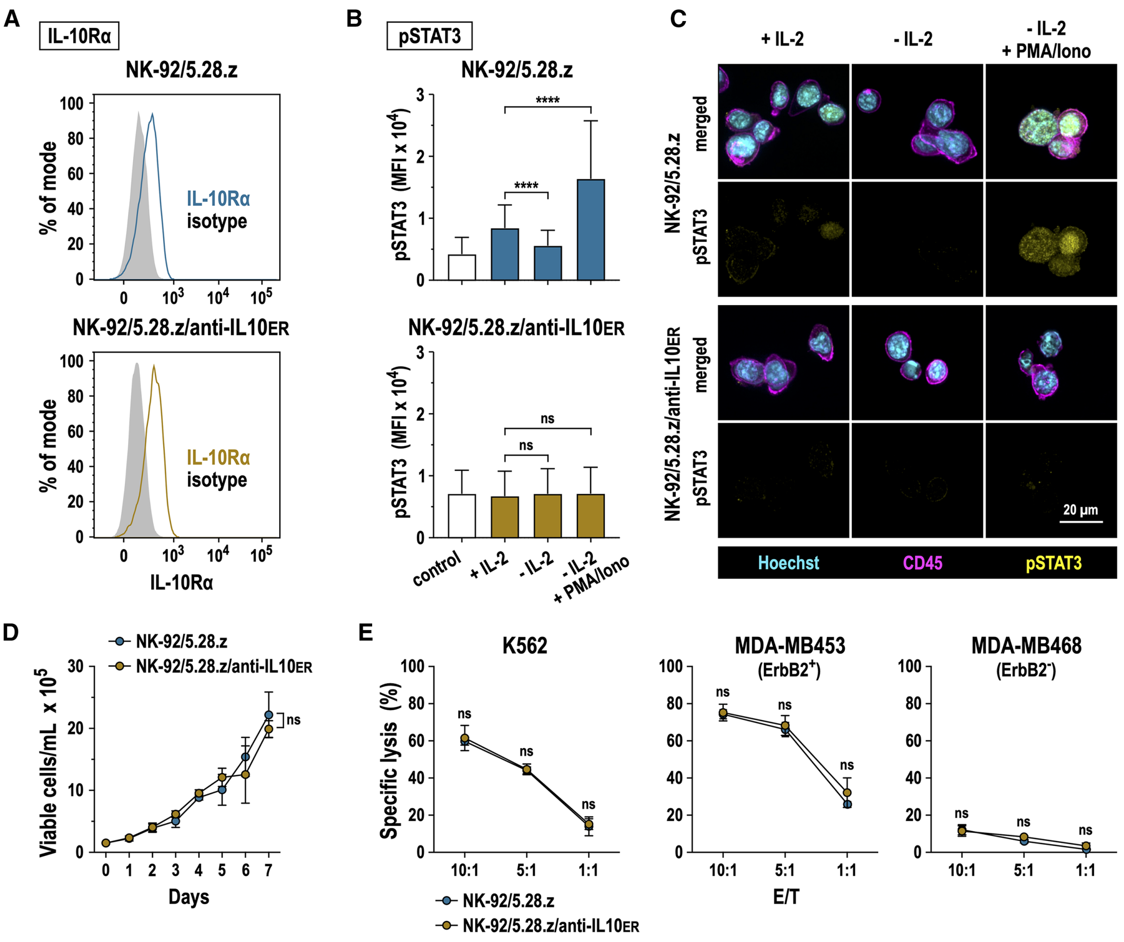

Figure 2. Functional analysis of NK-92/5.28.z/anti-IL10ER cells

(A) IL-10Rα expression on the surface of NK-92/5.28.z and NK-92/5.28.z/anti-IL10ER cells was analyzed by flow cytometry with fluorochrome-conjugated anti-IL-10Rα antibody (open areas). Controls were stained with an irrelevant antibody of the same isotype (filled gray areas). (B) IL-10 receptor downstream signaling was investigated by immunofluorescence microscopy detecting phosphorylated STAT3 (pSTAT3). NK-92/5.28.z and NK-92/5.28.z/anti-IL10ER cells were starved for 24 h in IL-2-free medium and then stimulated with PMA and ionomycin for 4 h to induce endogenous IL-10 expression (-IL-2/+PMA/Iono). Subsequently, the NK cells were fixed, permeabilized, and stained with APC-conjugated anti-CD45 antibody (magenta) and anti-pSTAT3 antibody followed by AF488-conjugated secondary antibody (yellow). Nuclei were stained with Hoechst dye (cyan). Cells kept in medium with (+IL-2) or without IL-2 (-IL-2) and cells only stained with secondary antibody (control) served as controls. Fluorescence signal quantification was performed with ImageJ by using a customized macro. Data are presented as mean ± SD of individual cells from at least three different images. ****$p < 0.0001$; ns (not significant), $p \geq 0.05$. Representative images are shown in (C). Scale bars, 20 μm. (D) Proliferation of NK-92/5.28.z and NK-92/5.28.z/anti-IL10ER cells. Cell counts were determined daily for 7 days, with dead cells excluded by trypan blue staining. Data are shown as mean ± SD from three independent experiments. ns (not significant), $p \geq 0.05$. (E) Cytotoxic activity of NK-92/5.28.z and NK-92/5.28.z/anti-IL10ER cells against K562 erythroleukemia and ErbB2-positive MDA-MB453 and ErbB2-negative MDA-MB468 breast carcinoma cells was investigated in flow cytometry-based assays after co-incubation with target cells for 2 hours at the indicated effector to target (E/T) ratios. Data are shown as mean ± SD from three independent experiments. ns, $p \geq 0.05$.

immunofluorescence microscopy data revealed modest amounts of phosphorylated STAT3 (pSTAT3) in NK-92/5.28.z cells maintained in IL-2-containing medium, likely due to the low level of IL-10 secreted by these cells under standard culture conditions (Figures 2B and 2C, top images). In contrast, in NK-92/5.28.z/anti-IL10ER cells, STAT3 was not phosphorylated in the presence of IL-2 (Figures 2B and 2C, bottom images). Upon stimulation with PMA and ionomycin, which strongly induced IL-10 production in NK-92/5.28.z (see Figures 1B and 1C), the unmodified CAR-NK cells exhibited markedly increased levels of pSTAT3, indicating autocrine stimulation by the secreted IL-10. No such effect was observed in NK-92/5.28.z/anti-IL10ER cells, which, despite activation, retained only basal levels of pSTAT3, demonstrating that intrabody-mediated retention of IL-10 effectively blocked autocrine signaling through the IL-10 receptor (Figures 2B and 2C, bottom images).

To test whether this blockade of IL-10 signaling in NK-92/5.28.z/anti-IL10ER cells affected their growth and functionality, we first examined proliferation over a 7-day period. Thereby, NK-92/5.28.z/anti-IL10ER and unmodified NK-92/5.28.z cells exhibited

very similar growth rates (Figure 2D), indicating that expression of the anti-IL-10 antibody and intracellular retention of IL-10 did not impair viability and proliferative potential. In addition, accumulation of the intrabody and trapped IL-10 in the ER did not result in ER stress, illustrated by the low expression of the ER chaperone GRP78/BIP, which remained at the level detected in unmodified NK-92/5.28.z cells (Figure S4A), and the absence of phosphorylated eukaryotic initiation factor 2α (phospho-eIF2α) (Figure S4B). Since STAT3 is implicated in regulating receptor expression in NK cells,[28] we also assessed expression of a large panel of activating receptors, inhibitory receptors, and exhaustion markers, including CD16, NKp30, NKp44, NKp46, NKp80, NKG2D, CD94, NKG2A, NKG2C, KIR2D, CTLA-4, LAG3, PD-L1, PD-1, TIM-3, and TIGIT, with no significant differences in their expression levels detected between NK-92/5.28.z/anti-IL10ER and unmodified NK-92/5.28.z cells (Figure S5). Next, we investigated potential effects of IL-10 depletion on cytotoxicity of NK-92/5.28.z/anti-IL10ER cells. Thereby, high natural cytotoxicity against K562 erythroleukemia and CAR-mediated lytic activity against ErbB2-positive MDA-MB453 breast cancer cells was observed, with no significant differences to unmodified NK-92/5.28.z cells (Figure 2E). This aligns well with the CAR-NK cells' similar activation-induced production of IFN-γ (see Figure S3). Neither NK-92/5.28.z/anti-IL10ER nor NK-92/5.28.z cells showed significant activity against ErbB2-negative MDA-MB468 breast cancer cells included as a control. Similar to NK-92/5.28.z/anti-IL10ER cells, proliferative capacity and natural cytotoxicity of parental NK-92 cells were not negatively affected if activation-induced secretion of IL-10 was blocked by anti-IL-10ER expression (Figures S2C and S2D).

Taken together, these data demonstrate that preventing IL-10 secretion has no adverse effects on viability or functionality of the NK-92/5.28.z/anti-IL10ER CAR-NK cells.

Effects of IL-10 depletion on bystander immune cells

As a pleiotropic cytokine, IL-10 exerts extensive regulatory effects on immune cells, capable of both, suppressing inflammation and modulating immune responses. To evaluate the influence of CAR-NK-derived IL-10 on bystander immune cells, we focused on DCs and macrophages, as these cells are critical in orchestrating an immune response and are highly sensitive to the immunomodulatory activity of IL-10. Flow-cytometric analysis of IL-10 receptor α (IL-10Rα) expression across peripheral blood mononuclear cell (PBMC) subsets confirmed its presence on the surface of multiple immune cell types, with particularly high levels found on monocyte subsets and DCs (Figures S6A–S6C). Significant expression of IL-10Rα was also detected on *in vitro* generated monocyte-derived DCs (MoDCs) and monocyte-derived macrophages (MDMs) (Figures S6D and S6E), underscoring their potential responsiveness to IL-10.

To assess the impact of IL-10 and other cytokines released by the engineered NK cells on MoDC maturation, we co-cultured human MoDCs with NK-92/5.28.z and NK-92/5.28.z/anti-IL10ER cells in the presence or absence of MDA-MB453 target cells using a transwell system, which allowed to examine the effects of secreted factors without direct cell-cell contact (Figure 3A). As expected, in control samples, recombinant TNF-α, IL-6, IL-1β, and prostaglandin E_2 (PGE2) readily induced maturation of MoDCs, indicated by an upregulation of the markers HLA-DR, CD83, and CD86, while this was not the case when MoDCs were cultivated with recombinant IL-10. Also, exposure to soluble factors from CAR-activated NK-92/5.28.z cells but not parental NK-92 cells lacking a CAR or NK cells in the absence of target cells induced a modest increase in the expression of the maturation markers HLA-DR and CD86 on MoDCs. Importantly, when MoDCs were instead exposed to CAR-activated NK-92/5.28.z/anti-IL10ER cells, the expression of maturation markers HLA-DR, CD86, and CD83 was far more pronounced, with statistically significant differences observed in both co-culture with parental NK-92 and unmodified NK-92/5.28.z cells. This demonstrates that cytokine secretion by CAR-activated NK cells can, to some extent, drive MoDC maturation, an effect that is substantially enhanced by preventing IL-10 release.

Next, we assessed the influence of the CAR-NK cells on MDM polarization. Exposure of unpolarized human MDMs in transwell co-cultures to factors secreted by NK-92/5.28.z cells after stimulation with ErbB2-positive MDA-MB453 target cells for CAR activation resulted in a significant decrease in HLA-DR and CD86 and an increase in CD163 expression in comparison with MDMs exposed to parental NK-92 cells (Figure 3B). This is consistent with polarization of the MDMs toward an M2-like, immunosuppressive phenotype. In sharp contrast, similar to culture in medium containing IFN-γ and lipopolysaccharide (LPS) driving polarization toward an M1-like phenotype, exposure of MDMs to soluble factors of activated NK-92/5.28.z/anti-IL-10ER cells not only led to a significant increase in HLA-DR and CD86 expression in comparison with NK-92 and NK-92/5.28.z cells but also prevented upregulation of the M2 marker CD163, which remained at baseline levels.

These findings indicate that activated NK-92/5.28.z cells favor polarization of neighboring macrophages toward an M2-like phenotype, which is undesired in the context of cancer immunotherapy. Importantly, depriving the CAR-NK cells of IL-10 by expression of the intracellular anti-IL-10ER antibody completely reversed this effect, and instead promoted polarization of human macrophages toward a pro-inflammatory M1-like phenotype.

In vivo antitumor activity of IL-10-depleted CAR-NK cells in an immunocompetent mouse glioma model

To assess whether blockade of IL-10 secretion affected *in vivo* antitumor activity of NK-92/5.28.z/anti-IL10ER cells, we employed an immunocompetent mouse glioma model based on GL261/ErbB2 cells, which stably express human ErbB2. With this model, we had previously demonstrated that treatment with NK-92/5.28.z CAR-NK cells in addition to direct antitumor effects can also activate endogenous antitumor immunity in syngeneic C57BL/6 mice.[15,16] As in the case of ErbB2-positive human MDA-MB453 breast cancer

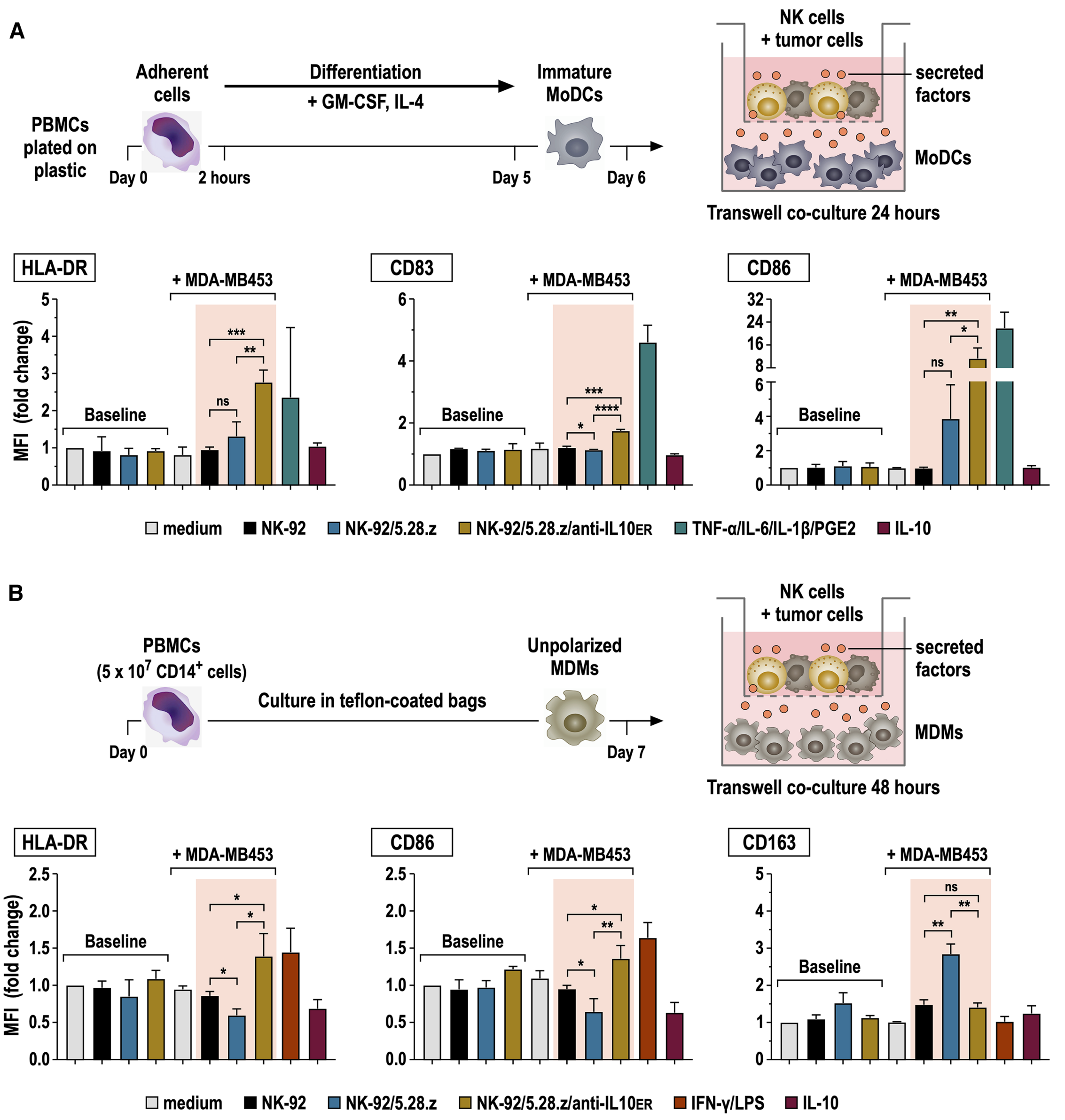

Figure 3. Influence of NK-92/5.28.z/anti-IL10ER cells on maturation and polarization of monocyte-derived dendritic cells and macrophages

(A) Human monocyte-derived dendritic cells (MoDCs) were generated by plating peripheral blood mononuclear cells (PBMCs) and removing non-adherent cells after 2 h. Adherent cells were cultured with GM-CSF and IL-4 from day 0 to day 5 to allow differentiation into immature MoDCs. On day 6, a transwell assay was set up with immature MoDCs in the lower chamber and CAR-engineered NK-92/5.28.z and NK-92/5.28.z/anti-IL10ER cells together with ErbB2-positive MDA-MB453 target cells at an effector to target ratio of 1:1 in the upper chamber (top). Parental NK-92 cells, MoDCs kept without NK cells, samples without tumor cells, MoDCs cultured with recombinant TNF-α, IL-6, IL-1β and prostaglandin E_2 (PGE2), or kept with recombinant IL-10 served as controls. After 24 h, flow cytometry was used to analyze surface expression of HLA-DR, CD83, and CD86 by the dendritic cells (bottom). (B) Human monocyte-derived macrophages (MDMs) were generated by seeding PBMCs into teflon-coated culture bags with medium replacement on days 2, 4, and 6, resulting in unpolarized MDMs by day 7. In the transwell setup, unpolarized MDMs were placed in the lower chamber, with NK-92/5.28.z and NK-92/5.28.z/anti-IL10ER cells together with MDA-MB453 target cells in the upper chamber as described in (A) (top). Parental NK-92 cells, MDMs cultured without NK cells, samples without tumor cells, MDMs cultured with recombinant IFN-γ and lipopolysaccharide (LPS), or kept with recombinant IL-10 served as controls. After 48 h, surface expression of HLA-DR, CD86, and CD163 by macrophages was assessed by flow cytometry (bottom). In (A) and (B), data are presented as relative mean fluorescent intensities (MFIs) normalized to untreated cells (baseline) ± SD from 3 independent donors. ****$p < 0.0001$; ***$p < 0.001$; **$p < 0.01$; *$p < 0.05$; ns, $p \geq 0.05$.

cells (see Figure 2E), the murine GL261/ErbB2 glioma cells triggered equally potent CAR-mediated cytotoxicity of NK-92/5.28.z/anti-IL10ER and unmodified NK-92/5.28.z cells *in vitro*, while ErbB2-negative GL261 control cells proved largely resistant to lysis by the CAR-NK cells (Figure 4A). Activation by GL261/ErbB2 cells also induced secretion of high amounts of IL-10 by NK-92/5.28.z cells,

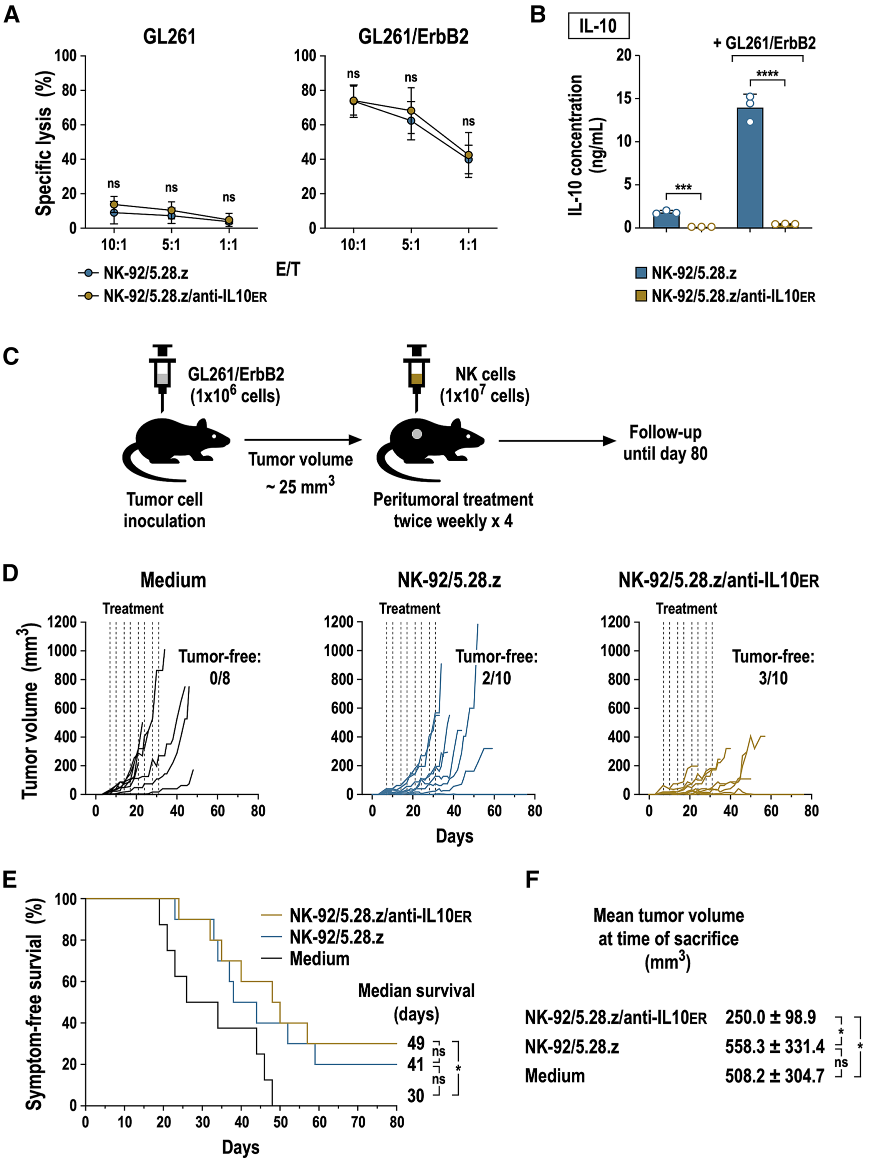

Figure 4. *In vivo* antitumor activity of NK-92/5.28.z/anti-IL10ER cells against syngeneic glioblastoma in immunocompetent C57BL/6 mice

(A) Cytotoxic activity of NK-92/5.28.z and NK-92/5.28.z/anti-IL10ER cells against GL261 and ErbB2-expressing GL261/ErbB2 murine glioblastoma cells was evaluated by flow cytometry after 2 h of co-culture at the indicated E/T ratios. Data represent mean values ±SD from three independent experiments. ns, $p \geq 0.05$. (B) IL-10 secretion by NK-92/5.28.z and NK-92/5.28.z/anti-IL-10ER cells at baseline and after CAR activation by exposure to GL261/ErbB2 cells was assessed using a cytometric bead array. Data are shown as mean ± SD from three independent experiments. ****$p < 0.0001$; ***$p < 0.001$. (C) Glioblastoma model to evaluate *in vivo* antitumor activity of CAR-NK cells. GL261/ErbB2 cells (1×10^6) were subcutaneously injected in syngeneic C57BL/6 mice. When tumors had reached an average volume of 25 mm^3, animals received peritumoral injections of 1×10^7 NK-92/5.28.z, NK-92/5.28.z/anti-IL10ER cells (n = 10), or medium (n = 8) twice weekly for 4 weeks, with follow-up until day 80. (D) Tumor growth in individual animals was followed by caliper measurements. (E) Symptom-free survival of the mice was monitored until day 80. Data were analyzed by Kaplan-Meier plot and log rank test. *$p < 0.05$; ns, $p \geq 0.05$. (F) Mean tumor volumes of tumor-bearing animals in the 3 treatment groups at time of sacrifice due to reaching a predefined stop criterion.

which was abrogated when the CAR-NK cells expressed the anti-IL-10ER intrabody (Figure 4B).

Next, we evaluated the therapeutic impact of IL-10-depleted NK-92/5.28.z/anti-IL10ER cells *in vivo*. GL261/ErbB2 tumor cells were injected subcutaneously into the flanks of C57BL/6 mice. Once tumors had engrafted and grown to an average volume of 25 mm^3, we initiated peritumoral treatment with either NK-92/5.28.z/anti-IL10ER or unmodified NK-92/5.28.z cells twice weekly for a total of 4 weeks (Figure 4C). While tumors grew progressively in all vehicle-treated control animals, 2 out of 10 mice that had received NK-92/5.28.z cells rejected their tumors and remained tumor-free until the end of the experiment at day 80 (Figure 4D). In mice treated with IL-10-depleted NK-92/5.28.z/anti-IL10ER cells, tumor growth kinetics were markedly delayed in comparison to treatment with unmodified CAR-NK cells, and 3 out of 10 mice completely rejected their tumors. Furthermore, survival analysis revealed a pronounced improvement in median symptom-free survival upon treatment with NK-92/5.28.z (41 days) and NK-92/5.28.z/anti-IL10ER cells (49 days) when compared with the medium control (30 days) (Figure 4E). Notably, this survival analysis underestimates the benefit of NK-92/5.28.z/anti-IL10ER cell treatment (see Figure 4D), as most of the tumor-bearing animals in this group had to be euthanized because of tumor ulceration indicative of pronounced local inflammation rather than a large tumor burden. This is evidenced by the significantly smaller mean tumor volume at the time of sacrifice (Figure 4F).

In summary, these findings demonstrate that NK-92/5.28.z/anti-IL10ER cells exhibit potent antitumor activity *in vivo*, with a more pronounced delay in tumor growth in the immunocompetent hosts than in animals treated with unmodified CAR-NK cells.

Influence of CAR-NK treatment on the tumor immune microenvironment

Due to the high sequence identity of human and murine IL-10, the human cytokine can readily bind to murine IL-10 receptors.[29,30]

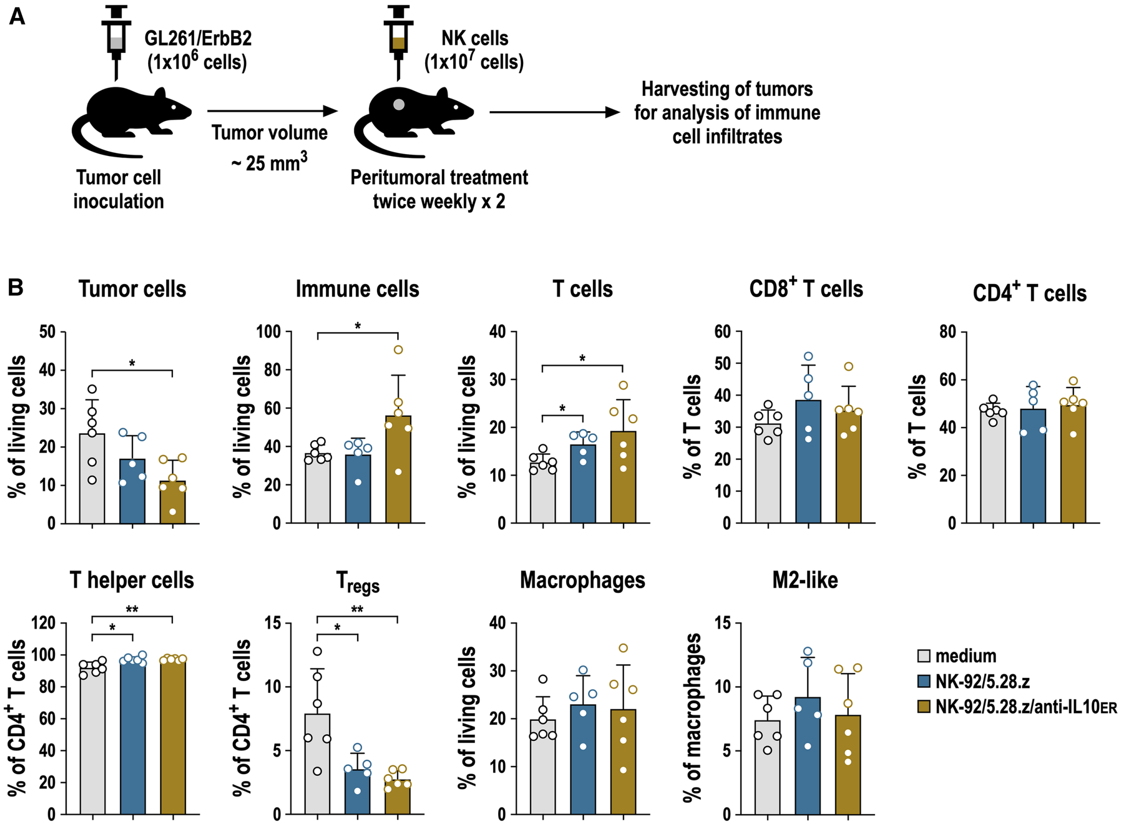

Figure 5. Flow-cytometric analysis of the tumor immune microenvironment in animals treated with NK-92/5.28.z and NK-92/5.28.z/anti-IL10ER cells
(A) C57BL/6 mice were subcutaneously injected with syngeneic GL261/ErbB2 cells and treated for 2 weeks with NK-92/5.28.z ($n = 5$), NK-92/5.28.z/anti-IL10ER cells ($n = 6$), or medium ($n = 6$) as described in Figure 4. (B) Tumors were excised one day after the final treatment, enzymatically dissociated, and single-cell suspensions were stained with fluorochrome-conjugated antibodies and a live/dead marker. The cellular composition was analyzed by flow cytometry and the proportions of the indicated cell subsets were quantified. Shown are means ± SD for tumor cells ($ErbB2^+$), immune cells ($CD45^+$), T cells ($CD45^+CD3^+$), $CD8^+$ T cells ($CD45^+CD3^+CD4^-CD8^+$), $CD4^+$ T cells ($CD45^+CD3^+CD4^+CD8^-$), T helper cells ($CD45^+CD3^+CD4^+CD8^-FoxP3^-$), regulatory T cells ($T_{reg}$ cells; $CD45^+CD3^+CD4^+CD8^-FoxP3^+$), macrophages ($CD45^+CD11b^+Ly6G^-F4.80^{high}Ly6C^{+/-}$), and macrophages expressing markers characteristic for an immunoregulatory phenotype ($CD45^+CD11b^+Ly6G^-F4.80^{high}Ly6C^{+/-}CD206^+$; for simplicity, indicated as M2-like). $**p < 0.01$; $*p < 0.05$.

Hence, IL-10 released by activated NK-92/5.28.z CAR-NK cells could exert suppressive effects on endogenous immune cells in C57BL/6 mice, which may be counteracted by the expression of the anti-IL-10ER intrabody. To test this hypothesis, we analyzed proportions and phenotype of infiltrating murine immune cell subsets in subcutaneous GL261/ErbB2 tumors upon treatment with NK-92/5.28.z or NK-92/5.28.z/anti-IL10ER cells. Similar to the experiment described earlier, tumor-bearing C57BL/6 mice received peritumoral injections of CAR-NK cells or injection medium twice weekly, but with treatment discontinued after 2 weeks and collection of tumor tissues done 1 day after the last treatment (Figure 5A).

In the first set of experiments, we performed flow-cytometric analysis of single-cell suspensions prepared from excised tumors. Thereby, treatment with both NK-92/5.28.z and NK-92/5.28.z/anti-IL10ER cells led to a reduction in the proportion of glioblastoma cells ($ErbB2^+$) in the tumor tissues compared with the medium control, which was more pronounced in the case of CAR-NK cells that expressed the anti-IL-10ER intrabody (Figure 5B). This was accompanied by a significant increase in infiltrating murine immune cells ($CD45^+$) upon treatment with NK-92/5.28.z/anti-IL10ER cells and enhanced numbers of total T cells ($CD3^+$) in the case of both CAR-NK derivatives (Figure 5B). While there were no statistically significant differences in infiltrating $CD8^+$ and total $CD4^+$ T cells between treatment groups, we noted a significant increase in T helper cells in comparison to the medium control and a concurrent reduction in regulatory T cells (T_{reg}; $CD4^+CD25^+FoxP3^+$) within the $CD4^+$ T cell populations after CAR-NK treatments, particularly in animals that had received NK-92/5.28.z/anti-IL10ER cells. Macrophage frequencies were not significantly different between the treatment groups in flow-cytometric analysis (Figure 5B). Nevertheless, there was a trend toward more macrophages carrying surface markers typical for a tumor-promoting phenotype ($CD45^+CD11b^+Ly6G^-F4.80^{high}Ly6C^{+/-}CD206^+$; for simplicity, indicated as M2-like) in

animals treated with unmodified NK-92/5.28.z cells, which was reversed when IL-10 secretion by the CAR-NK cells was blocked. In agreement with these findings, treatment with NK-92/5.28.z/anti-IL10ER cells induced a less pronounced tumor-specific serum antibody response than treatment with NK-92/5.28.z cells (Figure S7), arguing for predominant induction of a Th1 response by the IL-10-depleted CAR-NK cells.

To analyze treatment-induced modulation of the tumor immune microenvironment *in situ*, we also performed multiplex immunofluorescence microscopy of tumor sections. Representative microscopic images of an NK-92/5.28.z/anti-IL10ER-treated tumor are shown in Figure 6A, and quantitative analyses of tumors from all treatment groups are depicted in Figure 6B. Corroborating the findings from flow cytometry, CAR-NK treatments led to a reduction in tumor cell ($ErbB2^+$) density, with the NK-92/5.28.z/anti-IL10ER group showing the most pronounced decrease. Consistent with the flow cytometry data, we observed an increased T helper cell density and a reduced T_{reg} presence, particularly in NK-92/5.28.z/anti-IL10ER-treated tumors. Furthermore, NK-92/5.28.z/anti-IL10ER treatment promoted polarization of macrophages toward a more pro-inflammatory phenotype (for simplicity, indicated as M1-like) with significantly fewer macrophages carrying surface markers typical for a tumor-promoting phenotype ($CD68^+IL12^{low}CCR7^{low}CD163^{high}$ $CD206^{high}$; for simplicity, indicated as M2-like) compared with tumors treated with unmodified NK-92/5.28.z cells, confirming the trend seen in flow-cytometric analysis.

Collectively, these data demonstrate that both CAR-NK cell types reshaped the tumor immune microenvironment considerably. Thereby, NK-92/5.28.z/anti-IL10ER cells effectively reduced the numbers of IL-10-responsive immunosuppressive cells such as T_{reg} cells and immunoregulatory macrophages, promoting a more pro-inflammatory milieu.

DISCUSSION

In this study we investigated the consequences of preventing IL-10 release from clinical-grade NK-92/5.28.z cells for the CAR-NK cells' phenotype and functionality, and their crosstalk with bystander immune cells. Genome editing has been used to disrupt expression of undesired factors in various cell types and has also been employed successfully to modulate the functions of T lymphocytes and NK cells for cancer therapy.[31–34] Nevertheless, undesired off-target effects such as chromosome loss observed in CRISPR-Cas9-engineered T cells remain a considerable safety concern.[35–37] As an alternative approach, the expression of intrabodies allows direct interference with the target molecule on the protein level, thereby avoiding the risk of introducing genomic abnormalities.[38] In our study, we explored intracellular expression of an IL-10-specific scFv antibody as a means to prevent activation-induced secretion of the cytokine by CAR-NK cells. Following a strategy initially described for intracellular retention of viral glycoproteins and cell surface receptors,[26,39] we designed the antibody fragment to incorporate an N-terminal signal peptide to direct it to the secretory pathway and a C-terminal KDEL sequence to facilitate recognition by the cellular KDEL receptor for retrograde transport of the molecule from the Golgi complex and retention in the ER.[25,40]

Confocal microscopy confirmed effective ER retention of this anti-IL-10ER antibody together with IL-10 upon constitutive expression in ErbB2-specific NK-92/5.28.z CAR-NK cells, without inducing ER stress or affecting viability and proliferative capacity of the cells. This not only reduced IL-10 secretion at steady state to nearly undetectable levels, but also almost completely prevented activation-induced IL-10 release in the presence of CAR target cells. Importantly, while the loss of IL-10 secretion precluded autocrine activation of the IL-10 receptor pathway in NK-92/5.28.z/anti-IL10ER cells, this had no measurable consequences for natural and CAR-mediated cytotoxicity, nor did it alter cell surface expression of activating and inhibitory NK cell receptors or exhaustion markers previously described to be affected by STAT3 signaling, the main pathway downstream of the IL-10 receptor.[28] Hence, while IL-10 can enhance, to some extent, cytotoxic activity of primary NK cells against cancer cells,[41] this is different for CAR-engineered NK-92 cells, which retained full antitumor activity despite interruption of autocrine IL-10 signaling.

Notably, NK-92/5.28.z/anti-IL10ER cells produced more TNF-α than did unmodified NK-92/5.28.z cells upon CAR activation, while IFN-γ levels remained unaffected by depletion of IL-10. Given the opposing roles of IL-10 and TNF-α in regulating the functionality of DCs and other myeloid cells,[42] this likely contributed to the enhanced immunomodulatory activity of NK-92/5.28.z/anti-IL10ER cells observed in transwell co-culture assays with MoDCs and macrophages. IL-10 is known to negatively affect activation of these cell types, thereby suppressing their function as antigen-presenting cells.[43,44] Our data show that this was also the case for IL-10 secreted by unmodified NK-92/5.28.z cells upon CAR activation, which had a marked effect on the phenotype of co-cultured myeloid cells. While NK-92/5.28.z cells were, thereby, still able to stimulate DC maturation to some extent, this was significantly enhanced if IL-10 secretion was blocked. IL-10 released by unmodified CAR-NK cells was also responsible for polarization of co-cultured human macrophages toward an immunoregulatory M2-like phenotype undesirable in a tumor setting, since this effect was completely reversed upon intracellular expression of the anti-IL-10ER antibody, which instead promoted macrophage polarization toward a pro-inflammatory M1-like phenotype.

We previously demonstrated therapeutic efficacy of repeated local injections of NK-92/5.28.z CAR-NK cells in murine models of ErbB2-positive glioblastoma,[15] with this treatment approach also adopted for the ongoing CAR2BRAIN phase I clinical trial in glioblastoma patients (NCT03383978).[9,45] Thereby, NK-92/5.28.z therapy facilitated complete tumor rejection and protection from subsequent tumor rechallenge in syngeneic glioblastoma models in immunocompetent animals.[15] This highlights the dual mode of

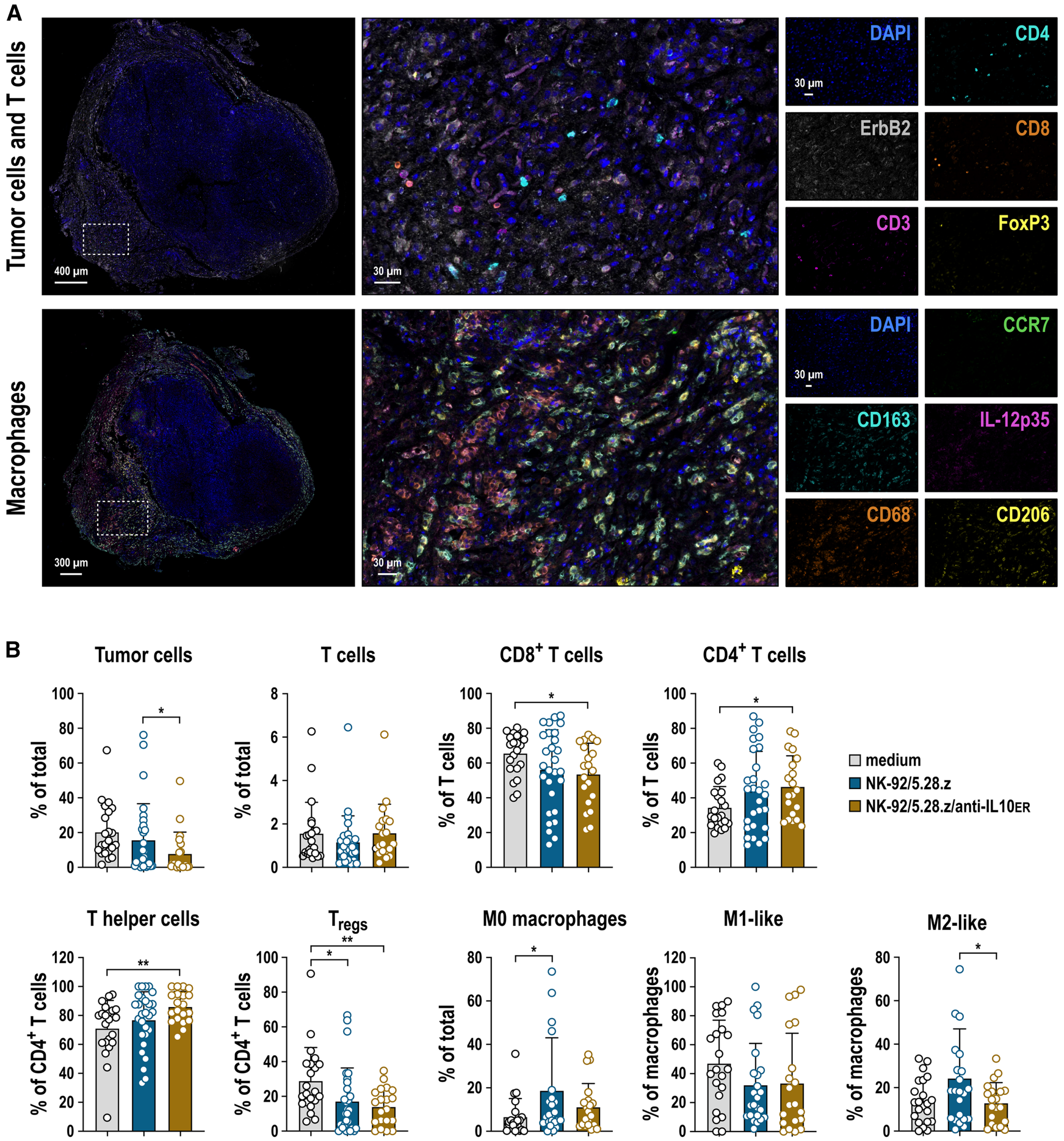

Figure 6. *In situ* analysis of the tumor immune microenvironment in animals treated with NK-92/5.28.z and NK-92/5.28.z/anti-IL10ER cells

(A) Representative composite images of a GL261/ErbB2 tumor harvested after treatment with NK-92/5.28.z/anti-IL10ER cells, depicting multiplex immunofluorescence stainings for ErbB2 (light gray), CD3 (magenta), CD4 (cyan), CD8 (orange), and FoxP3 (yellow) to identify tumor cells and T cell subpopulations (top) or for macrophage markers CD163 (cyan), CD68 (orange), CCR7 (green), IL-12p35 (magenta), and CD206 (yellow) (bottom). DAPI was used to detect nuclei (blue). Whole slide scans are depicted on the left, with the positions of zoomed-in regions shown in middle and right, highlighted. Scale bars are indicated in the images. (B) Quantitative analysis of tumor cell and immune cell populations as a proportion of the indicated subsets, performed across all treatment groups using multiplex stainings and automated image analysis. Shown are means ± SD for tumor cells ($ErbB2^+$), total T cells ($CD3^+$), $CD8^+$ T cells ($CD3^+CD8^+$), $CD4^+$ T cells ($CD3^+CD4^+$), T helper cells ($CD3^+CD4^+FoxP3^-$), regulatory T cells (T_{reg} cells; $CD3^+CD4^+FoxP3^+$), pro-inflammatory macrophages ($CD68^+$IL-12^{high} and $CCR7^{high}CD163^{low}CD206^{low}$; for simplicity, indicated as M1-like), macrophages expressing markers characteristic for an immunoregulatory phenotype ($CD68^+$IL-$12^{low}CCR7^{low}CD163^{high}CD206^{high}$; for simplicity, indicated as M2-like), and M0 macrophages (all other $CD68^+CD163^+$ cells). Data points represent quantitative data from different microscopic fields of n = 3–4 tumors per treatment group. $^{**}p < 0.01$; $^{*}p < 0.05$.

action of the human CAR-NK cells, which, in addition to CAR-mediated direct cytotoxicity, can also stimulate endogenous antitumor immunity even in mice, as evidenced by the induction of a tumor-specific humoral immune response and T-cell-mediated long-term protection from tumor rechallenge in animals cured after NK-92/5.28.z therapy.[15,16] Likewise, in this study, treatment of

syngeneic GL261/ErbB2 tumors in immunocompetent C57BL/6 mice with NK-92/5.28.z and NK-92/5.28.z/anti-IL10ER cells resulted in a marked inhibition of tumor growth, with complete tumor rejection in a subset of animals. Thereby, NK-92/5.28.z/anti-IL10ER cells delayed tumor progression more effectively than unmodified NK-92/5.28.z cells and resulted in a longer symptom-free survival, despite more pronounced tumor inflammation in the NK-92/5.28.z/anti-IL10ER group. Notably, some of the NK-92/5.28.z/anti-IL10ER-treated animals had to be prematurely sacrificed according to animal welfare regulations due to tumor ulceration, although they carried only small tumors. Possibly, these tumors may otherwise have been rejected in the course of the ongoing immune reaction. To limit adverse effects, the possibility of such an increased inflammatory response will have to be considered when determining dosing and treatment schedule for potential clinical translation of IL-10-depleted NK-92/5.28.z cells.

Importantly, treatment with NK-92/5.28.z/anti-IL10ER cells more effectively reshaped the tumor immune microenvironment in the immunocompetent GL261/ErbB2 mouse glioma model than did NK-92/5.28.z cells, thus considerably reducing infiltration by IL-10-responsive immunosuppressive cell populations like T_{reg} cells and immunoregulatory macrophages, accompanied by a shift toward a more pro-inflammatory milieu. Human IL-10 is biologically active in mice,[29,30] which enabled us to examine the consequences of preventing IL-10 release from the human CAR-NK cells *in vivo*. Still, the use of NK-92/5.28.z and NK-92/5.28.z/anti-IL10ER cells in an immunocompetent murine model constitutes a limitation of our study. It is noteworthy, however, that the blockade of IL-10 secretion in NK-92/5.28.z/anti-IL10ER cells resulted in similar effects in the murine tumors *in vivo* as seen in the co-culture experiments with human immune cells *in vitro*, most notably the prevention of macrophage polarization toward an immunosuppressive phenotype otherwise induced by unmodified NK-92/5.28.z cells. Furthermore, unlike IL-10, key pro-inflammatory cytokines like IFN-γ and TNF-α secreted by activated human CAR-NK cells are not or only partially active in mice.[46,47] This raises the possibility that elimination of immunoregulatory IL-10 from CAR-NK cells, which could otherwise counteract these pro-inflammatory factors, may exert even more pronounced immunomodulatory effects in a human tumor setting. While we did not detect significant upregulation of IL-10 expression in *ex vivo* expanded primary NK cells after activation (Figure S8), blockade of IL-10 secretion strongly enhanced the therapeutic utility of the clinical-grade NK-92/5.28.z cells investigated in this study, and may, therefore, be highly relevant also for other CAR-engineered NK-92 derivatives currently undergoing active clinical development.[10]

Taken together, our data demonstrate that immunosuppressive IL-10 produced by CAR-engineered NK-92 cells contributes significantly to their crosstalk with neighboring immune cells, thereby limiting their ability to activate endogenous immune effectors in the tumor microenvironment and recruit them for a concerted anti-tumor response. Preventing IL-10 secretion in NK-92/5.28.z cells effectively abrogated these undesired effects and markedly enhanced the pro-inflammatory and immunomodulatory activity of the ErbB2-specific CAR-NK cells. The dose escalation part of our ongoing phase I clinical trial in glioblastoma has already demonstrated the feasibility and safety of administering NK-92/5.28.z cells to cancer patients. This may aid further development of a potentially more efficacious IL-10-depleted variant of the off-the-shelf therapeutic and accelerate clinical translation of this promising approach.

MATERIALS AND METHODS

Cells and culture conditions

Human MDA-MB453 and MDA-MB468 breast carcinoma cells and murine GL261 and GL261/ErbB2 glioblastoma cells were cultured in DMEM (Gibco, Thermo Fisher Scientific, Darmstadt, Germany). K562 erythroleukemia cells were maintained in RPMI (Gibco). Media were supplemented with 10% heat-inactivated fetal bovine serum (FBS) (Capricorn Scientific, Ebsdorfergrund, Germany), 2 mM L-glutamine, 100 U/mL penicillin, and 100 μg/mL streptomycin (all from Gibco). NK-92 cells (kindly provided by NantKwest, Inc., Culver City, CA)[48] and CAR-engineered variants were cultured in X-VIVO 10 (Lonza, Cologne, Germany) supplemented with 5% heat-inactivated human AB plasma (Red Cross Blood Donation Service Baden-Württemberg-Hessen, Frankfurt, Germany) and 100 IU/mL IL-2 (Proleukin, Novartis Pharma, Nürnberg, Germany).

Expression of ER-retained anti-IL-10 antibody

The ER-retained anti-IL-10 antibody (anti-IL-10ER) was designed *in silico*, incorporating an immunoglobulin heavy chain signal peptide, an scFv domain of humanized IL-10-specific antibody BT-063,[49] an influenza virus HA tag, and a KDEL ER retention sequence.[26] Codon-optimized anti-IL-10ER cDNA was generated by *de novo* synthesis (GeneArt, Thermo Fisher Scientific) and inserted upstream of internal ribosome entry site (IRES) and EGFP sequences in lentiviral transfer vector pHR'SIN-cPPT-SIEW (pSIEW),[50] resulting in plasmid pS-anti-IL10ER-IEW. Lentiviral particles were generated and used to transduce NK-92/5.28.z cells as described.[51] These NK-92/5.28.z CAR-NK cells were previously generated as a GMP-compliant single-cell clone by transducing NK-92 cells with a lentiviral vector encoding an ErbB2-specific second-generation CAR with CD28 costimulatory and CD3ζ signaling domains.[19] EGFP-expressing NK-92/5.28.z/anti-IL10ER cells carrying both constructs were enriched by flow-cytometric cell sorting with a FACSAria Fusion device (BD Biosciences, Heidelberg, Germany), and expression of the anti-IL-10ER antibody was confirmed by intracellular staining with AF647-conjugated HA-tag-specific antibody (16B12; BioLegend, Koblenz, Germany) and flow-cytometric analysis using a FACSCanto II cytometer (BD Biosciences). For detection of anti-IL-10ER by immunoblot analysis, whole cell lysates (25 μg of total proteins per lane) were separated by SDS-PAGE under reducing conditions, transferred to polyvinylidene fluoride (PVDF) membranes, and incubated with HA-tag-specific antibody (C29F4; Cell Signaling Technology, Leiden, The Netherlands), followed by HRP-conjugated secondary antibody and chemiluminescent detection. As a loading control, γ-tubulin was

detected with a polyclonal antibody (Sigma-Aldrich, Merck, Darmstadt, Germany).

Subcellular localization of anti-IL-10ER and IL-10 was examined by microscopic analysis. NK cells were stimulated with 5 ng/mL PMA and 50 ng/mL ionomycin (both from Sigma-Aldrich) for 4 h, fixed with methanol, and stained with AF647-conjugated anti-HA-tag (16B12), PE-Dazzle594-conjugated anti-IL-10 (JES3-9D7) (both from BioLegend), and AF488-conjugated anti-calreticulin (EPR3924; Abcam, Cambridge, United Kingdom) antibodies. Nuclei were counterstained with Hoechst 33342 dye (Thermo Fisher Scientific). Microscopic images were acquired with a CQ1 Confocal Quantitative Image Cytometer (Yokogawa, Tokyo, Japan). For colocalization analysis, single z stacks were processed with ImageJ software (v.1.54k; National Institutes of Health, Bethesda, MD; https://imagej.net/ij/), and individual cells in six different fields were analyzed using the EzColocalization plug-in to quantify PCC and generate scatterplots.[52]

To examine viability and proliferation of anti-IL-10ER-expressing NK cells, 1.5×10^5 cells were seeded in 1 mL culture medium, and cell counts were determined daily for a period of 7 days, with dead cells excluded by trypan blue staining. Expression of ER stress proteins was assessed by immunoblot analysis. 2×10^6 NK cells were seeded in 3 mL culture medium and incubated overnight. NK cells treated with 0.5 μM thapsigargin (Sigma-Aldrich) served as positive control. Whole cell lysates (25 μg of total proteins per lane) were separated by SDS-PAGE under reducing conditions, transferred to PVDF membranes, and probed with antibodies specific for GRP78/BIP (C50B12), phospho-eIF2α (D9G8), or total eIF2α (D7D3) (all from Cell Signaling Technology), followed by HRP-conjugated secondary antibodies and chemiluminescent detection. γ-tubulin served as loading control.

Analysis of cytokine expression

IL-10 mRNA expression was analyzed by quantitative real-time PCR. Total RNA from NK cells was isolated using the RNeasy Plus Mini Kit (Qiagen, Hilden, Germany) according to the manufacturer's instructions, followed by cDNA synthesis with M-MuLV reverse transcriptase (New England Biolabs, Frankfurt am Main, Germany), RiboLock RNase Inhibitor (Thermo Fisher Scientific), oligo(dT) primer, and dNTPs (New England Biolabs). The resulting cDNA served as template for qPCR on a LightCycler 480 (Roche, Basel, Switzerland) using the Mesa Green qPCR MasterMix Plus (Eurogentec, Lüttich, Belgium) with customized primers. Relative mRNA expression was calculated using the $2^{-\Delta\Delta CT}$ method, with β_2 microglobulin (β2m) as reference gene. Forward and reverse primers used were, respectively, 5′ AAGACCCAGACATCAAGGCG 3′ and 5′ CACGGCCTTGCTCTTGTTTT 3' for IL-10 and 5′ ATGAGTATGC CTGCCGTGTGA 3′ and 5′ GGCATCTTCAAACCTCCATG 3' for β2m. PCRs were performed in triplicates, including controls without cDNA.

IL-10 protein expression was examined by intracellular antibody staining. NK cells were stimulated in the presence of GolgiStop (BD Biosciences) either with 1 μg/mL PMA and 1 μg/mL ionomycin or by co-incubation with target cells at an effector to target (E/T) ratio of 1:1. After a 3 h incubation, cells were harvested, stained with BV421-conjugated anti-CD56 antibody (NCAM16.2; BD Biosciences), fixed, and permeabilized using the BD Cytotox/Cytoperm Kit (BD Biosciences), followed by staining with PE-conjugated anti-IL-10 antibody (JES3-9D7; BioLegend) and flow-cytometric analysis. Cytokine release by NK-92 cells was measured using a BD CBA and a BD FACSArray bioanalyzer (BD Biosciences) according to the manufacturer's recommendations. Briefly, 5×10^5 NK-92 cells were co-cultured for 6 h at 37°C with target cells at an E/T ratio of 1:1 in a total volume of 1 mL. Cytokine concentrations in supernatants were determined using BD CBA Flex Sets for IFN-γ, IL-10, MIP-1α/CCL3 and TNF-α (all from BD Biosciences), and data were analyzed with BD FCAP Array software (BD Biosciences).

IL-10 receptor expression and signaling

Expression of IL-10Rα on the surface of NK-92 cells was analyzed by flow cytometry with APC-conjugated anti-IL-10Rα antibody (3F9; BioLegend), including a matched isotype control. IL-10Rα and lineage marker expression on PBMCs from healthy donors was assessed by flow cytometry with PE-conjugated anti-IL-10Rα (3F9), BV711-conjugated anti-CD11c (B-ly6), BV786-conjugated anti-CD16 (3G8), AF700-conjugated anti-CD19 (HIB19) (all from BD Biosciences), APC-conjugated anti-CD11b (M1/70.15.11.5; Miltenyi Biotec, Bergisch Gladbach, Germany), APC-Fire-conjugated anti-CD3 (UCHT1), BV510-conjugated anti-CD4 (A161A1), BV421-conjugated anti-CD8 (SK1), FITC-conjugated anti-CD14 (HCD14), and PE-Cy7-conjugated anti-CD56 (5.1H11) (all from BioLegend) antibodies using an LSRFortessa Cell Analyzer (BD Biosciences). Prior to staining, PBMCs were incubated with True-Stain Monocyte Blocker (BioLegend).

IL-10-mediated downstream signaling was assessed by detecting pSTAT3 via immunofluorescence microscopy. NK cells were cultured in IL-2-containing medium or starved for 24 h before stimulation with 5 ng/mL PMA and 50 ng/mL ionomycin for 4 h to induce endogenous IL-10 expression. Cells were fixed with methanol and permeabilized using 0.3% Triton X-100 in DPBS, followed by incubation overnight at 4°C with anti-pSTAT3 antibody (D3A7; Cell Signaling Technology) and subsequent staining with AF488-conjugated secondary antibody and APC-conjugated anti-CD45 (2D1; eBioscience, Thermo Fisher Scientific) antibody. Nuclei were counterstained with Hoechst 33342 dye. Images were acquired with a CQ1 Confocal Quantitative Image Cytometer and analyzed by using ImageJ software with a customized macro to quantify mean fluorescence intensities of the pSTAT3 signal in individual cells (see Method S1).

Analysis of NK cell activity

Surface expression of NK cell receptors and exhaustion markers by NK-92/5.28.z and NK-92/5.28.z/anti-IL10ER cells was analyzed by flow cytometry with an LSRFortessa cell analyzer after staining of cells with fluorochrome-conjugated antibodies specific for CD16

(3G8), NKp44 (p44-8) (both from BD Biosciences), NKp30 (REA823), NKp46 (9E2), NKp80 (4A4.D10), CD94 (REA113), KIR2D (NKVFS1), NKG2A (REA110), NKG2C (REA205), NKG2D (BAT221) (all from Miltenyi Biotec), CTLA-4 (L3D10), LAG-3 (7H2C65), PD-1 (EH12.2H7), PD-L1 (w), TIM-3 (F38-2E2) (all from BioLegend), and TIGIT (MBSA43; Invitrogen, Thermo Fisher Scientific).

Cytolytic activity of NK cells was analyzed in flow cytometry-based cytotoxicity assays. Target cells were labeled with Calcein Violet AM (CV) (CellTrace, Invitrogen, Thermo Fisher Scientific) and co-cultured with effector cells at different E/T ratios for 2 h at 37°C. After co-incubation, 100 μL of propidium iodide (PI) solution (1 μg/mL) was added to each sample, and cells were analyzed with a FACSCanto II flow cytometer. CV and PI double-positive cells were identified as dead target cells. To calculate specific cytotoxicity, spontaneous target cell lysis in the absence of NK cells was subtracted. Data were analyzed using FlowJo software (v.10.9.0; BD Biosciences).

Transwell co-culture assays with dendritic cells and macrophages

The effects of soluble factors secreted by CAR-NK cells on co-cultured human immune cells were investigated in transwell assays with MoDCs and MDMs. PBMCs from healthy donors were isolated from buffy coats by Ficoll density gradient centrifugation. For the generation of MoDCs, 2×10^6 PBMCs/cm^2 were plated in 10 cm culture dishes with RPMI medium containing 5% heat-inactivated human AB plasma, 2 mM L-glutamine, 100 U/mL penicillin, and 100 μg/mL streptomycin. Following 2 h of incubation at 37°C, non-adherent cells were removed; the remaining adherent cells were washed, and fresh RPMI medium supplemented with 50 ng/mL granulocyte-macrophage colony-stimulating factor (GM-CSF) and 50 ng/mL IL-4 (both from PeproTech, Hamburg, Germany) was added. Cells were cultured for 5 days to allow differentiation into immature MoDCs, with a medium change on day 3. Then, 5×10^5 immature MoDCs were seeded in 24-well plates with medium containing GM-CSF and IL-4. One day later, MoDCs were either used in transwell assays or stimulated for 24 h with 10 ng/mL IL-1β, 25 ng/mL IL-6, 10 ng/mL TNF-α (all from PeproTech), and 1 μg/mL PGE2 (Sigma Aldrich) to induce maturation.

For MDM generation, PBMCs were first stained with anti-CD14 antibody (MϕP9; BD Biosciences) and analyzed by flow cytometry with an LSRFortessa Cell Analyzer to determine the percentage of CD14-positive monocytes. Then, a total of 5×10^7 CD14-positive cells were seeded in PermaLife Cell Culture bags (OriGen Biomedical, Austin, TX) in RPMI medium supplemented with 5% heat-inactivated human AB plasma, 2 mM L-glutamine, 100 U/mL penicillin, and 100 μg/mL streptomycin, with medium changes every 2 days. After 7 days, MDMs were transferred to 6-well dishes (Nunc, Thermo Fisher Scientific) for use in transwell assays or allowed to polarize into M1-like macrophages by adding 50 ng/mL IFN-γ and 10 ng/mL LPS or into M2-like macrophages by adding 20 ng/mL IL-10.

In the transwell setup, 4×10^5 to 5×10^5 primary immune cells (MoDCs or MDMs) were seeded in the lower chamber of a 6-well culture plate. Transwell inserts (ThinCert, 0.4 μm pore size; Greiner Bio-One, Frickenhausen, Germany) were used to separate the primary immune cells from NK and tumor cells, which were added to the upper chamber with 1×10^6 cells each. After incubation for 24 or 48 h at 37°C, transwell inserts were removed, the MoDCs or MDMs were harvested from the lower chambers, and cells were examined by flow cytometry for surface marker expression. MoDCs were analyzed after staining with BV711-conjugated anti-CD83 (HB15e), APC-conjugated anti-CD86 (2331), and BV510-conjugated anti-HLA-DR (G46-6) antibodies (all from BD Biosciences). MDMs were stained with FITC-conjugated anti-CD163 (GHI/61; BD Biosciences) antibody and the same anti-CD86 and anti-HLA-DR antibodies as aforementioned.

Animal experiments

The *in vivo* antitumor activity of NK cells was assessed in a syngeneic GL261/ErbB2 subcutaneous glioblastoma model. 6 to 8 weeks old female C57BL/6 mice (Charles River, Sulzfeld, Germany) were inoculated with 1×10^6 tumor cells at the right flank. When tumors had reached an average volume of 25 mm^3, animals were treated twice weekly by peritumoral injection of 1×10^7 NK-92/5.28.z or NK-92/5.28.z/anti-IL10ER cells for 2 to 4 weeks. Control animals received injection medium. Tumor growth was followed by caliper measurements at preset intervals, and tumor volumes were calculated using the formula: length $\times$ (width)^2 $\times$ 0.5. Health status and symptom-free survival were assessed by daily inspection. Group sizes were chosen to allow statistical analysis of differences in symptom-free survial. All treated animals were included in the analysis. Animal experiments were approved by the responsible government committee (Regierungspräsidium Darmstadt, Darmstadt, Germany; protocol number F123/1033), and were conducted in the animal facility of Georg-Speyer-Haus according to all applicable guidelines and regulations.

Immunophenotyping of GL261/ErbB2 tumors

For flow-cytometric analysis of immune cell infiltrates, subcutaneous GL261/ErbB2 tumors were harvested one day after the final treatment, tissues were minced, and enzymatically dissociated under orbital shaking (130 rpm) at 37°C in Advanced DMEM/F12 medium (Gibco) supplemented with 50 U/mL collagenase (Sigma-Aldrich). Enzymatic dissociation was supported by pipetting the mixtures up and down every 20–30 min. After 60 min, 20 U/mL DNase (Sigma Aldrich) was supplemented, and the samples were incubated for another 30 min before cells were collected by centrifugation. Single-cell suspensions were then prepared for staining with specific antibody panels. Panel 1 included PerCP-conjugated anti-CD45 (30-F11) and AF647-conjugated ErbB2-specific (24D2) antibodies (both from BioLegend). Panel 2 consisted of APC-conjugated anti-CD4 (RM4-5) and FITC-conjugated anti-CD25 (3C7) antibodies (both from BD Biosciences) and PE-Cy7-conjugated anti-CD3 (17A2), PerCP-Cy5.5-conjugated anti-CD8α (53-6.7), BV510-conjugated anti-CD45 (30-F11), and BV421-conjugated anti-FoxP3

(MF-14) antibodies (all from BioLegend). Panel 3 included PE-Vio770-conjugated anti-CD11b (M1/70.15.11.5; Miltenyi Biotec) and AF647-conjugated anti-CD206 (MR5D3; BD Biosciences) as well as BV510-conjugated anti-CD45 (30-F11), PE-conjugated anti-F4/80 (BM8), BV421-conjugated anti-Ly6C (HK1.4), and PerCP-conjugated anti-Ly6G (1A8) antibodies (all from BioLegend). All panels also included Fixable Viability Dye eFluor 780 (Thermo Fisher Scientific). Analyses were performed with a FACSCanto II flow cytometer.

For multiplex immunofluorescence microscopy, tumors harvested one day after the final treatment were fixed in 4% PFA, dehydrated using an ASP300S device (Leica Biosystems, Wetzlar, Germany), embedded in paraffin, and sectioned (3 μm). Sections were deparaffinized and stained with Opal 7-Color Automation IHC Kits (Akoya Biosciences, Marlborough, MA) through a BOND-RX Multiplex IHC Stainer (Leica Biosystems). Each section was put through 6 sequential rounds of staining, which included blocking in 1× Antibody Diluent/Block (Akoya Biosciences), followed by incubation with either anti-CD3ε (SP7; Abcam), anti-CD4 (D7D2Z; Cell Signaling Technology), anti-CD8α (D4W2Z; Cell Signaling Technology), anti-ErbB2 (CL0268; Atlas Antibodies, Stockholm, Sweden), and anti-FoxP3 (D6O8R; Cell Signaling Technology) antibodies (panel 1), or anti-CCR7 (ERP23192-57; Abcam), anti-CD68 (PG-M1; DAKO, Agilent Technologies, Waldbronn, Germany), anti-CD163 (EPR19518; Abcam), anti-CD206 (polyclonal; Abcam), and anti-IL-12p35 (polyclonal; Thermo Fisher Scientific) antibodies (panel 2), corresponding secondary HRP-conjugated antibodies and Opal fluorophores as described.[53] Nuclei were counterstained with 4′,6-diamidino-2-phenylindole (DAPI) contained in the Opal 7-Color Automation IHC Kits, and slides were mounted with Fluoromount-G (SouthernBiotech, Birmingham, AL). Images were acquired with the PhenoImager HT imaging system (Akoya Biosciences) and analyzed using the phenotyping application of the inForm software V2.4.10 (Akoya Biosciences).

Statistical analysis

Data were analyzed using unpaired Student's *t* test. Symptom-free survival was evaluated by Kaplan-Meier plot and log rank (Mantel-Cox) test. p values <0.05 were considered statistically significant and are indicated as follows: $^{****}p < 0.0001$; $^{***}p < 0.001$; $^{**}p < 0.01$; $^{*}p < 0.05$. Prism 10 software (v.10.1.1.323; GraphPad Software, Boston, MA) was used for all statistical calculations.

DATA AND CODE AVAILABILITY

Data supporting the findings of this study are available within the article, the supplemental information, or from the corresponding author upon reasonable request.

ACKNOWLEDGMENTS

We thank Pranav Oberoi for kindly providing peripheral blood-derived primary NK cells, Barbara Uherek and Thorsten Geyer for technical assistance, Annette Klemke for flow-cytometric cell sorting, Petra Schön for help with cytometric bead arrays, Petra Dinse for preparation of tissue slides, and the staff at the animal facility of Georg-Speyer-Haus for their support. This work was supported in part by grant no. WE 2589/6-1 from Deutsche Forschungsgemeinschaft (DFG) and institutional funds of Georg-Speyer-Haus. Georg-Speyer-Haus is funded jointly by the German Federal Ministry of Health and the Hessian Ministry of Science and Research, Arts and Culture.

AUTHOR CONTRIBUTIONS

A.L., J.R., C.Z., and W.S.W. designed the study; A.L., J.R., A.H., A.B., M.M., N.M., M.S., A.K., and I.K. performed the experiments; A.L., J.R., A.H., M.M., A.W., and W.S.W. analyzed data; T.T., M.K., S.S., and C.Z. provided critical input and reagents; J.R., A.L., and W.S.W wrote the manuscript. All authors contributed to the critical review and editing of the manuscript and approved the final version.

DECLARATION OF INTERESTS

A.L., T.T., M.K., C.Z., and W.S.W. are named as inventors on patents and patent applications in the field of CAR-NK cells and cancer immunotherapy owned by their respective academic institutions.

SUPPLEMENTAL INFORMATION

Supplemental information can be found online at https://doi.org/10.1016/j.omton.2026.201285.

REFERENCES

1. Weber, E.W., Maus, M.V., and Mackall, C.L. (2020). The emerging landscape of immune cell therapies. Cell *181*, 46–62. https://doi.org/10.1016/j.cell.2020.03.001.

2. Pahl, J., and Cerwenka, A. (2017). Tricking the balance: NK cells in anti-cancer immunity. Immunobiology *222*, 11–20. https://doi.org/10.1016/j.imbio.2015.07.012.

3. Shimasaki, N., Jain, A., and Campana, D. (2020). NK cells for cancer immunotherapy. Nat. Rev. Drug Discov. *19*, 200–218. https://doi.org/10.1038/s41573-019-0052-1.

4. Maskalenko, N.A., Zhigarev, D., and Campbell, K.S. (2022). Harnessing natural killer cells for cancer immunotherapy: dispatching the first responders. Nat. Rev. Drug Discov. *21*, 559–577. https://doi.org/10.1038/s41573-022-00413-7.

5. Liu, E., Marin, D., Banerjee, P., Macapinlac, H.A., Thompson, P., Basar, R., Nassif Kerbauy, L., Overman, B., Thall, P., Kaplan, M., et al. (2020). Use of CAR-transduced natural killer cells in CD19-positive lymphoid tumors. N. Engl. J. Med. *382*, 545–553. https://doi.org/10.1056/NEJMoa1910607.

6. Marin, D., Li, Y., Basar, R., Rafei, H., Daher, M., Dou, J., Mohanty, V., Dede, M., Nieto, Y., Uprety, N., et al. (2024). Safety, efficacy and determinants of response of allogeneic CD19-specific CAR-NK cells in CD19(+) B cell tumors: a phase 1/2 trial. Nat. Med. *30*, 772–784. https://doi.org/10.1038/s41591-023-02785-8.

7. Ghobadi, A., Bachanova, V., Patel, K., Park, J.H., Flinn, I., Riedell, P.A., Bachier, C., Diefenbach, C.S., Wong, C., Bickers, C., et al. (2025). Induced pluripotent stem-cell-derived CD19-directed chimeric antigen receptor natural killer cells in B-cell lymphoma: a phase 1, first-in-human trial. Lancet *405*, 127–136. https://doi.org/10.1016/s0140-6736(24)02462-0.

8. Tang, X., Yang, L., Li, Z., Nalin, A.P., Dai, H., Xu, T., Yin, J., You, F., Zhu, M., Shen, W., et al. (2018). First-in-man clinical trial of CAR NK-92 cells: safety test of CD33-CAR NK-92 cells in patients with relapsed and refractory acute myeloid leukemia. Am. J. Cancer Res. *8*, 1083–1089.

9. Burger, M.C., Forster, M.T., Romanski, A., Straßheimer, F., Macas, J., Zeiner, P.S., Steidl, E., Herkt, S., Weber, K.J., Schupp, J., et al. (2023). Intracranial injection of natural killer cells engineered with a HER2-targeted chimeric antigen receptor in patients with recurrent glioblastoma. Neuro Oncol. *25*, 2058–2071. https://doi.org/10.1093/neuonc/noad087.

10. Kühnel, I., Tonn, T., and Wels, W.S. (2026). Engineered natural killer cell lines and their application for cancer immunotherapy. In Natural Killer Cells: At the Forefront of Modern Immunology, J. Zimmer and E. Ullrich, eds. (Springer Berlin Heidelberg), pp. 1–30. https://doi.org/10.1007/978-3-662-68816-8_36-2.

11. Wagner, J., Wickman, E., DeRenzo, C., and Gottschalk, S. (2020). CAR T cell therapy for solid tumors: Bright future or dark reality? Mol. Ther. *28*, 2320–2339. https://doi.org/10.1016/j.ymthe.2020.09.015.

12. Vivier, E., Rebuffet, L., Narni-Mancinelli, E., Cornen, S., Igarashi, R.Y., and Fantin, V.R. (2024). Natural killer cell therapies. Nature *626*, 727–736. https://doi.org/10.1038/s41586-023-06945-1.

13. Walzer, T., Dalod, M., Robbins, S.H., Zitvogel, L., and Vivier, E. (2005). Natural-killer cells and dendritic cells: "l'union fait la force". Blood *106*, 2252–2258. https://doi.org/10.1182/blood-2005-03-1154.

14. Ferlazzo, G., and Morandi, B. (2014). Cross-talks between natural killer cells and distinct subsets of dendritic cells. Front. Immunol. *5*, 159. https://doi.org/10.3389/fimmu.2014.00159.

15. Zhang, C., Burger, M.C., Jennewein, L., Genßler, S., Schönfeld, K., Zeiner, P., Hattingen, E., Harter, P.N., Mittelbronn, M., Tonn, T., et al. (2016). ErbB2/HER2-specific NK cells for targeted therapy of glioblastoma. J. Natl. Cancer Inst. *108*, djv375. https://doi.org/10.1093/jnci/djv375.

16. Zhang, C., Oberoi, P., Oelsner, S., Waldmann, A., Lindner, A., Tonn, T., and Wels, W.S. (2017). Chimeric antigen receptor-engineered NK-92 cells: An off-the-shelf cellular therapeutic for targeted elimination of cancer cells and induction of protective antitumor immunity. Front. Immunol. *8*, 533. https://doi.org/10.3389/fimmu.2017.00533.

17. Strecker, M.I., Wlotzka, K., Strassheimer, F., Roller, B., Ludmirski, G., König, S., Röder, J., Opitz, C., Alekseeva, T., Reul, J., et al. (2022). AAV-mediated gene transfer of a checkpoint inhibitor in combination with HER2-targeted CAR-NK cells as experimental therapy for glioblastoma. Oncoimmunology *11*, 2127508. https://doi.org/10.1080/2162402x.2022.2127508.

18. Strassheimer, F., Elleringmann, P., Ludmirski, G., Roller, B., Macas, J., Alekseeva, T., Cakmak, P., Aliraj, B., Krenzlin, H., Demes, M.C., et al. (2025). CAR-NK cell therapy combined with checkpoint inhibition induces an NKT cell response in glioblastoma. Br. J. Cancer *132*, 849–860. https://doi.org/10.1038/s41416-025-02977-8.

19. Schönfeld, K., Sahm, C., Zhang, C., Naundorf, S., Brendel, C., Odendahl, M., Nowakowska, P., Bönig, H., Köhl, U., Kloess, S., et al. (2015). Selective inhibition of tumor growth by clonal NK cells expressing an ErbB2/HER2-specific chimeric antigen receptor. Mol. Ther. *23*, 330–338. https://doi.org/10.1038/mt.2014.219.

20. Röder, J., Alekseeva, T., Kiefer, A., Kühnel, I., Prüfer, M., Zhang, C., Bodden, M., Rosigkeit, S., Waldmann, A., Tonn, T., et al. (2025). ErbB2/HER2-targeted CAR-NK cells eliminate breast cancer cells in an organoid model that recapitulates tumor progression. Mol. Ther. *33*, 3559–3575. https://doi.org/10.1016/j.ymthe.2025.04.033.

21. Nowakowska, P., Romanski, A., Miller, N., Odendahl, M., Bönig, H., Zhang, C., Seifried, E., Wels, W.S., and Tonn, T. (2018). Clinical grade manufacturing of genetically modified, CAR-expressing NK-92 cells for the treatment of ErbB2-positive malignancies. Cancer Immunol. Immunother. *67*, 25–38. https://doi.org/10.1007/s00262-017-2055-2.

22. Blois, S.M., Freitag, N., Tirado-González, I., Cheng, S.B., Heimesaat, M.M., Bereswill, S., Rose, M., Conrad, M.L., Barrientos, G., and Sharma, S. (2017). NK cell-derived IL-10 is critical for DC-NK cell dialogue at the maternal-fetal interface. Sci. Rep. *7*, 2189. https://doi.org/10.1038/s41598-017-02333-8.

23. Perona-Wright, G., Mohrs, K., Szaba, F.M., Kummer, L.W., Madan, R., Karp, C.L., Johnson, L.L., Smiley, S.T., and Mohrs, M. (2009). Systemic but not local infections elicit immunosuppressive IL-10 production by natural killer cells. Cell Host Microbe *6*, 503–512. https://doi.org/10.1016/j.chom.2009.11.003.

24. Ostapchuk, Y.O., Cetin, E.A., Perfilyeva, Y.V., Yilmaz, A., Skiba, Y.A., Chirkin, A.P., Omarbaeva, N.A., Talaeva, S.G., Belyaev, N.N., and Deniz, G. (2015). Peripheral blood NK cells expressing HLA-G, IL-10 and TGF-β in healthy donors and breast cancer patients. Cell. Immunol. *298*, 37–46. https://doi.org/10.1016/j.cellimm.2015.09.002.

25. Munro, S., and Pelham, H.R. (1987). A C-terminal signal prevents secretion of luminal ER proteins. Cell *48*, 899–907. https://doi.org/10.1016/0092-8674(87)90086-9.

26. Beerli, R.R., Wels, W., and Hynes, N.E. (1994). Intracellular expression of single chain antibodies reverts ErbB-2 transformation. J. Biol. Chem. *269*, 23931–23936.

27. Wang, Z., Guan, D., Huo, J., Biswas, S.K., Huang, Y., Yang, Y., Xu, S., and Lam, K.P. (2021). IL-10 enhances human natural killer cell effector functions via metabolic reprogramming regulated by mTORC1 signaling. Front. Immunol. *12*, 619195. https://doi.org/10.3389/fimmu.2021.619195.

28. Witalisz-Siepracka, A., Klein, K., Zdársky, B., and Stoiber, D. (2022). The multifaceted role of STAT3 in NK-cell tumor surveillance. Front. Immunol. *13*, 947568. https://doi.org/10.3389/fimmu.2022.947568.

29. Tan, J.C., Indelicato, S.R., Narula, S.K., Zavodny, P.J., and Chou, C.C. (1993). Characterization of interleukin-10 receptors on human and mouse cells. J. Biol. Chem. *268*, 21053–21059.

30. Zdanov, A., Schalk-Hihi, C., Gustchina, A., Tsang, M., Weatherbee, J., and Wlodawer, A. (1995). Crystal structure of interleukin-10 reveals the functional dimer with an unexpected topological similarity to interferon gamma. Structure *3*, 591–601. https://doi.org/10.1016/s0969-2126(01)00193-9.

31. Biederstädt, A., Manzar, G.S., and Daher, M. (2022). Multiplexed engineering and precision gene editing in cellular immunotherapy. Front. Immunol. *13*, 1063303. https://doi.org/10.3389/fimmu.2022.1063303.

32. Georgiadis, C., Preece, R., and Qasim, W. (2025). Clinical development of allogeneic chimeric antigen receptor αβ-T cells. Mol. Ther. *33*, 2426–2440. https://doi.org/10.1016/j.ymthe.2025.03.040.

33. Petri, K., D'Ippolito, E., Künkele, A., Köhl, U., Busch, D.H., Einsele, H., and Hudecek, M. (2025). Next-generation T cell immunotherapies engineered with CRISPR base and prime editing: challenges and opportunities. Nat. Rev. Clin. Oncol. *22*, 902–923. https://doi.org/10.1038/s41571-025-01072-4.

34. Bexte, T., Albinger, N., Al Ajami, A., Wendel, P., Buchinger, L., Gessner, A., Alzubi, J., Särchen, V., Vogler, M., Rasheed, H.M., et al. (2024). CRISPR/Cas9 editing of NKG2A improves the efficacy of primary CD33-directed chimeric antigen receptor natural killer cells. Nat. Commun. *15*, 8439. https://doi.org/10.1038/s41467-024-52388-1.

35. Kalter, N., Fuster-García, C., Silva, A., Ronco-Díaz, V., Roncelli, S., Turchiano, G., Gorodkin, J., Cathomen, T., Benabdellah, K., Lee, C., and Hendel, A. (2025). Off-target effects in CRISPR-Cas genome editing for human therapeutics: Progress and challenges. Mol. Ther. Nucleic Acids *36*, 102636. https://doi.org/10.1016/j.omtn.2025.102636.

36. Nahmad, A.D., Reuveni, E., Goldschmidt, E., Tenne, T., Liberman, M., Horovitz-Fried, M., Khosravi, R., Kobo, H., Reinstein, E., Madi, A., et al. (2022). Frequent aneuploidy in primary human T cells after CRISPR-Cas9 cleavage. Nat. Biotechnol. *40*, 1807–1813. https://doi.org/10.1038/s41587-022-01377-0.

37. Tsuchida, C.A., Brandes, N., Bueno, R., Trinidad, M., Mazumder, T., Yu, B., Hwang, B., Chang, C., Liu, J., Sun, Y., et al. (2023). Mitigation of chromosome loss in clinical CRISPR-Cas9-engineered T cells. Cell *186*, 4567–4582.e20. https://doi.org/10.1016/j.cell.2023.08.041.

38. Zhang, C., Ötjengerdes, R.M., Roewe, J., Mejias, R., and Marschall, A.L.J. (2020). Applying antibodies inside cells: Principles and recent advances in neurobiology, virology and oncology. BioDrugs *34*, 435–462. https://doi.org/10.1007/s40259-020-00419-w.

39. Marasco, W.A., Haseltine, W.A., and Chen, S.Y. (1993). Design, intracellular expression, and activity of a human anti-human immunodeficiency virus type 1 gp120 single-chain antibody. Proc. Natl. Acad. Sci. USA *90*, 7889–7893. https://doi.org/10.1073/pnas.90.16.7889.

40. Lewis, M.J., and Pelham, H.R. (1992). Ligand-induced redistribution of a human KDEL receptor from the Golgi complex to the endoplasmic reticulum. Cell *68*, 353–364. https://doi.org/10.1016/0092-8674(92)90476-s.

41. Mocellin, S., Panelli, M., Wang, E., Rossi, C.R., Pilati, P., Nitti, D., Lise, M., and Marincola, F.M. (2004). IL-10 stimulatory effects on human NK cells explored by gene profile analysis. Genes Immun. *5*, 621–630. https://doi.org/10.1038/sj.gene.6364135.

42. Sato, K., Nagayama, H., Tadokoro, K., Juji, T., and Takahashi, T.A. (1999). Extracellular signal-regulated kinase, stress-activated protein kinase/c-Jun N-terminal kinase, and p38mapk are involved in IL-10-mediated selective repression of TNF-alpha-induced activation and maturation of human peripheral blood monocyte-derived dendritic cells. J. Immunol. *162*, 3865–3872.

43. Sato, T., Terai, M., Tamura, Y., Alexeev, V., Mastrangelo, M.J., and Selvan, S.R. (2011). Interleukin 10 in the tumor microenvironment: a target for anticancer immunotherapy. Immunol. Res. *51*, 170–182. https://doi.org/10.1007/s12026-011-8262-6.

44. Mittal, S.K., and Roche, P.A. (2015). Suppression of antigen presentation by IL-10. Curr. Opin. Immunol. *34*, 22–27. https://doi.org/10.1016/j.coi.2014.12.009.

45. Burger, M.C., Zhang, C., Harter, P.N., Romanski, A., Strassheimer, F., Senft, C., Tonn, T., Steinbach, J.P., and Wels, W.S. (2019). CAR-engineered NK cells for the

treatment of glioblastoma: Turning innate effectors into precision tools for cancer immunotherapy. Front. Immunol. *10*, 2683. https://doi.org/10.3389/fimmu.2019.02683.

46. Hemmi, S., Merlin, G., and Aguet, M. (1992). Functional characterization of a hybrid human-mouse interferon gamma receptor: evidence for species-specific interaction of the extracellular receptor domain with a putative signal transducer. Proc. Natl. Acad. Sci. USA *89*, 2737–2741. https://doi.org/10.1073/pnas.89.7.2737.

47. Ameloot, P., Fiers, W., De Bleser, P., Ware, C.F., Vandenabeele, P., and Brouckaert, P. (2001). Identification of tumor necrosis factor (TNF) amino acids crucial for binding to the murine p75 TNF receptor and construction of receptor-selective mutants. J. Biol. Chem. *276*, 37426–37430. https://doi.org/10.1074/jbc.M102020200.

48. Gong, J.H., Maki, G., and Klingemann, H.G. (1994). Characterization of a human cell line (NK-92) with phenotypical and functional characteristics of activated natural killer cells. Leukemia *8*, 652–658.

49. Osterroth, F., Uherek, C., Bruecher, C., Rottgen, P., Daelken, B., Engling, A., Zuber, C., Czeloth, N., Wartenberg-Demand, A., Gutscher, M., and Wessels-Kranz, J. (2012). Humanized anti-IL-10 antibodies for the treatment of systemic lupus erythematosus (SLE). United States patent application US2012/0321617A1. filed November 30, 2010, and granted February 17, 2015.

50. Demaison, C., Parsley, K., Brouns, G., Scherr, M., Battmer, K., Kinnon, C., Grez, M., and Thrasher, A.J. (2002). High-level transduction and gene expression in hematopoietic repopulating cells using a human immunodeficiency virus type 1-based lentiviral vector containing an internal spleen focus forming virus promoter. Hum. Gene Ther. *13*, 803–813. https://doi.org/10.1089/10430340252898984.

51. Sahm, C., Schönfeld, K., and Wels, W.S. (2012). Expression of IL-15 in NK cells results in rapid enrichment and selective cytotoxicity of gene-modified effectors that carry a tumor-specific antigen receptor. Cancer Immunol. Immunother. *61*, 1451–1461. https://doi.org/10.1007/s00262-012-1212-x.

52. Stauffer, W., Sheng, H., and Lim, H.N. (2018). EzColocalization: An ImageJ plugin for visualizing and measuring colocalization in cells and organisms. Sci. Rep. *8*, 15764. https://doi.org/10.1038/s41598-018-33592-8.

53. Strack, E., Rolfe, P.A., Fink, A.F., Bankov, K., Schmid, T., Solbach, C., Savai, R., Sha, W., Pradel, L., Hartmann, S., et al. (2020). Identification of tumor-associated macrophage subsets that are associated with breast cancer prognosis. Clin. Transl. Med. *10*, e239. https://doi.org/10.1002/ctm2.239.

OMTON, Volume *34*

Supplemental information

Depletion of IL-10 in CAR-NK cells augments reprogramming of the tumor microenvironment and ameliorates therapeutic efficacy

Anja Löwe, Jasmin Röder, Aline Häcker, Anita Bhatti, Margarete Mijatovic, Nina Müller, Malena Schnieder, Anne Kiefer, Ines Kühnel, Torsten Tonn, Manuel Kaulich, Stefan Stein, Congcong Zhang, Andreas Weigert, and Winfried S. Wels

Supplemental Figures

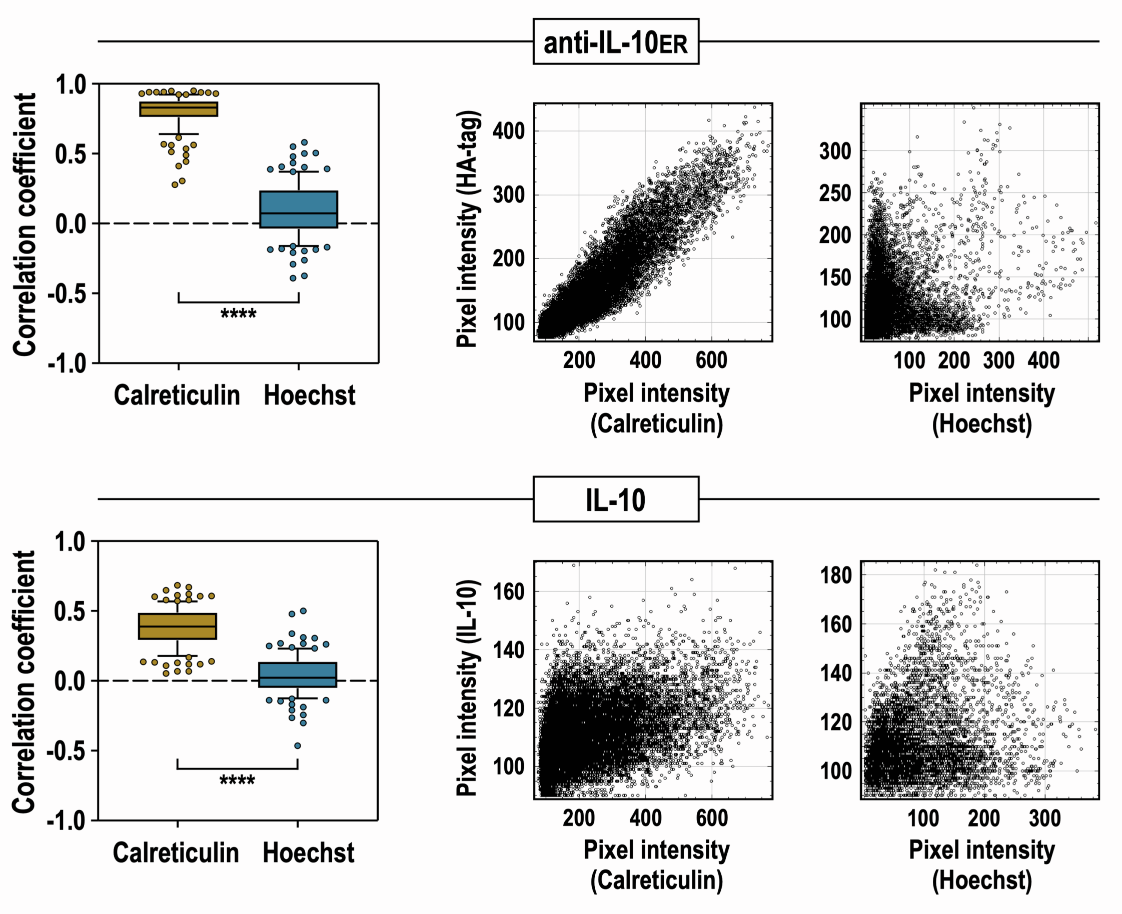

Figure S1. Colocalization of anti-IL-10ER antibody and IL-10 in the endoplasmic reticulum of NK-92/5.28.z/anti-IL10ER cells

NK-92/5.28.z/anti-IL10ER cells were activated by stimulation with PMA and ionomycin, fixed, permeabilized, and stained with antibodies specific for intracellular anti-IL-10ER antibody (HA-tag), calreticulin, and IL-10. Nuclei were stained with Hoechst dye. Images were processed using a customized macro, and colocalization analysis was conducted with the EzColocalization plugin in ImageJ. Left: Colocalization analysis is displayed as Pearson's correlation coefficients (PCC) between anti-IL-10ER and calreticulin or Hoechst (upper panel), or IL-10 and calreticulin or Hoechst (lower panel). PCC values were calculated for individual cells and summarized as whisker box plots representing the 10th–90th percentiles. Data were derived from six images per group. Right: Representative scatter plots, where each pixel's intensity in each channel is plotted, illustrating the degree of colocalization. ****, $p<0.0001$.

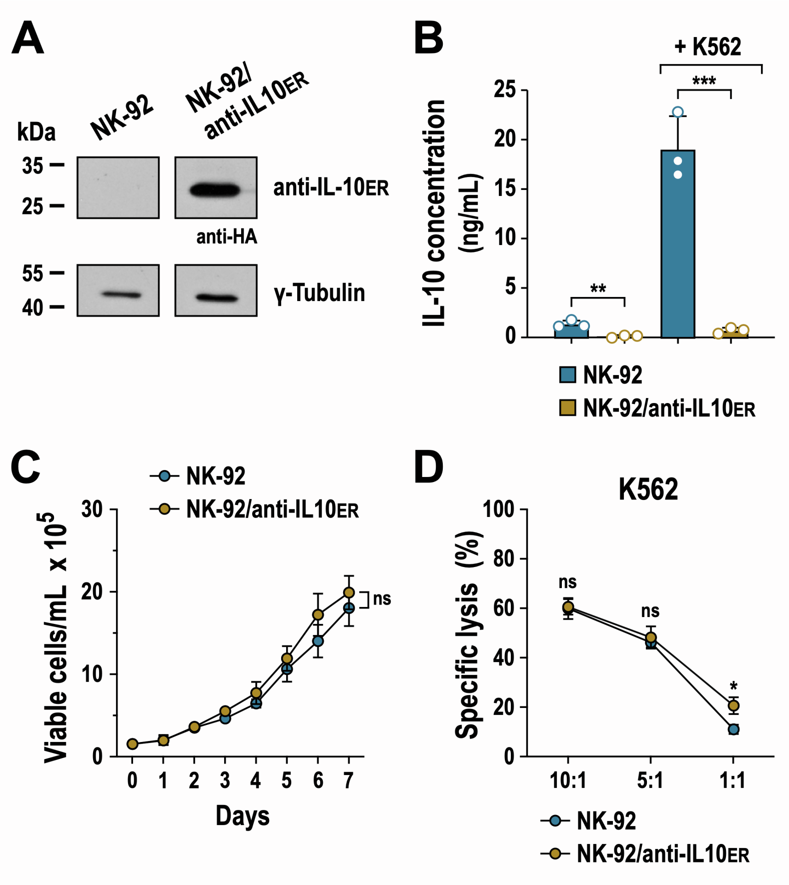

Figure S2. Expression of intracellular anti-IL-10 antibody in parental NK-92 cells
(A) Immunoblot analysis of whole cell lysates of NK-92 and NK-92/anti-IL10ER cells generated by lentiviral transduction of parental NK-92 cells with anti-IL-10ER-encoding vector as described in the main text for CAR-positive NK-92/5.28.z cells. The anti-IL-10ER molecule was detected with HA-tag-specific antibody. γ-tubulin served as a loading control. (B) IL-10 secretion by NK-92 and NK-92/anti-IL10ER cells at steady state and following activation by co-incubation with K562 target cells for 6 hours at an effector to target (E/T) ratio of 1:1 was assessed using a cytometric bead array. Data are shown as mean ± SD from three independent experiments. ***, $p < 0.001$; **, $p < 0.01$. (C) Proliferation of NK-92 and NK-92/anti-IL10ER cells. Cell counts were determined daily for 7 days, with dead cells excluded by trypan blue staining. Data are shown as mean ± SD from three independent experiments. ns (not significant), $p \geq 0.05$. (D) Cytotoxic activity of NK-92 and NK-92/anti-IL10ER cells against K562 erythroleukemia cells was investigated in flow cytometry-based assays after co-incubation with target cells for 2 hours at the indicated effector to target (E/T) ratios. Data are shown as mean ± SD from three independent experiments. *, $p < 0.05$; ns, $p \geq 0.05$.

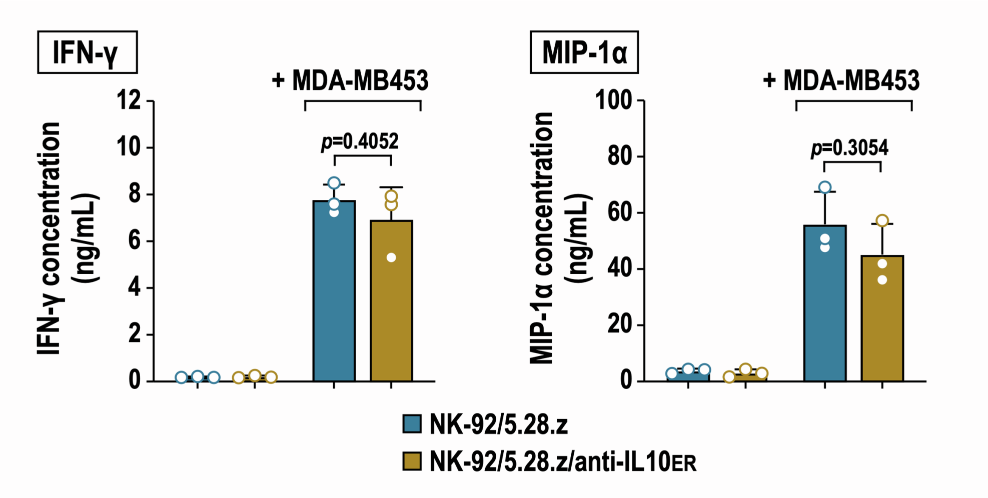

Figure S3. Effects of anti-IL-10ER expression on cytokine production
IFN-γ (left) and MIP-1α secretion (right) by NK-92/5.28.z and NK-92/5.28.z/anti-IL10ER cells at steady state and following CAR activation by co-incubation with ErbB2-positive MDA-MB453 target cells for 6 hours at an effector to target (E/T) ratio of 1:1 was assessed using a cytometric bead array. Data are shown as mean ± SD from three independent experiments.

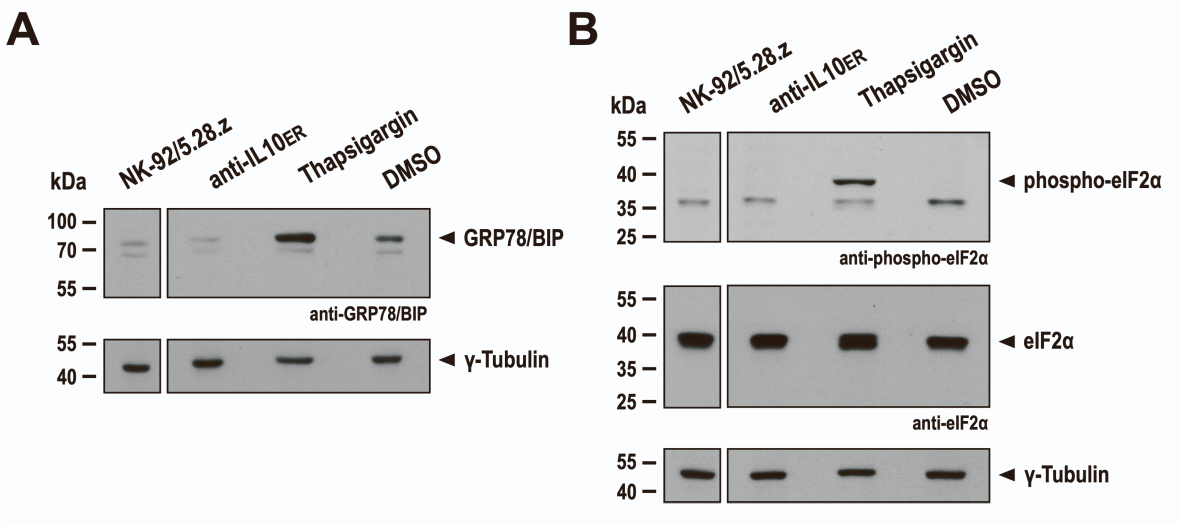

Figure S4. Analysis of ER stress
Whole cell lysates of NK-92/5.28.z and NK-92/5.28.z/anti-IL10ER cells were analyzed by SDS-PAGE and immunoblotting using antibodies specific for the ER chaperone GRP78/BIP (A), or phosphorylated eIF2α (phospho- eIF2α) (B) as markers of ER stress. eIF2α and γ-tubulin served as loading controls. NK-92/5.28.z cells treated with thapsigargin or DMSO were used as controls for ER stress induction.

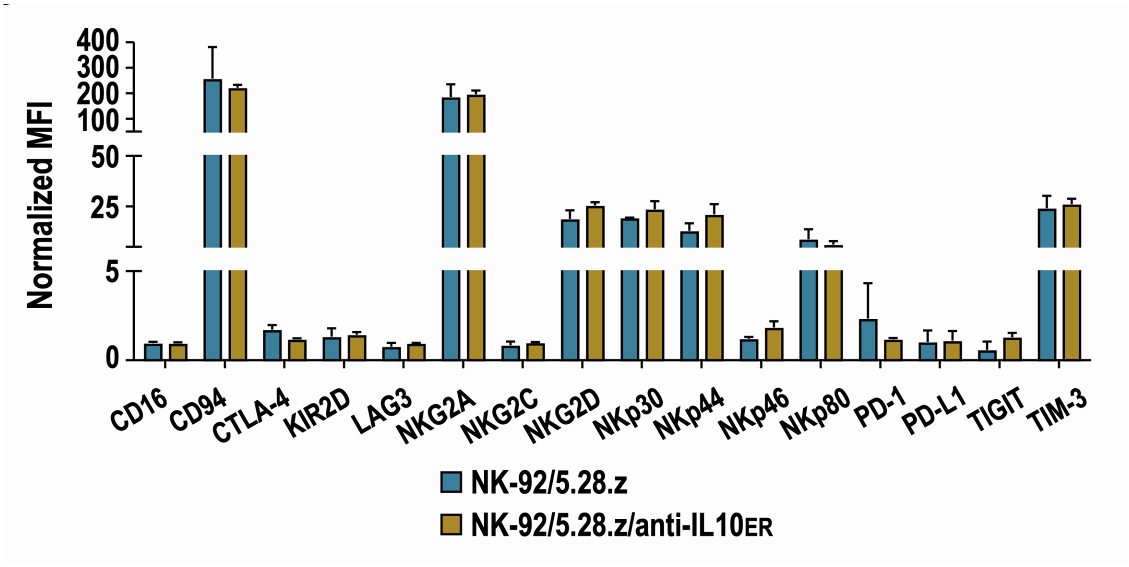

Figure S5. Effects of anti-IL-10ER expression on surface marker expression
Surface expression of the indicated activating and inhibitory NK cell receptors and exhaustion markers on NK-92/5.28.z and NK-92/5.28.z/anti-IL10ER cells was analyzed by staining with specific antibodies and flow cytometry. Mean fluorescence intensities were normalized to unstained controls and are presented as mean values ± SD from three independent experiments.

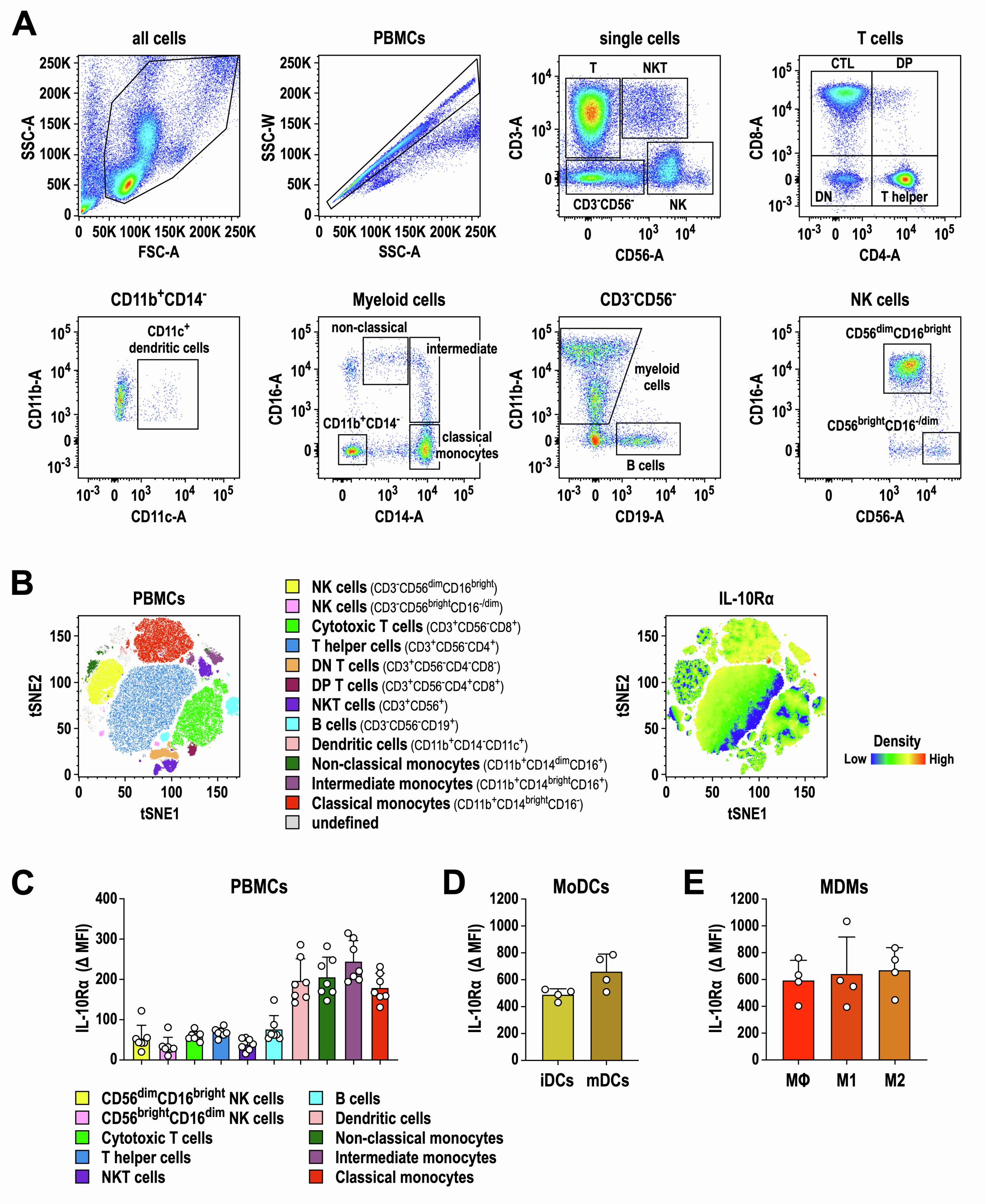

Figure S6. IL-10Rα expression on immune cell subsets

(A) PBMCs from healthy donors were stained with fluorochrome-conjugated antibodies specific for CD3, CD4, CD8, CD11b, CD11c, CD14, CD16, CD19, and CD56 for identification of key immune cell subsets by multicolor flow cytometry. Shown is the gating strategy used to define each subset: T helper cells ($CD3^+CD4^+CD56^-$), cytotoxic T cells (CTL; $CD3^+CD8^+CD56^-$), double-positive T cells (DP; $CD3^+CD4^+CD8^+$), double negative T cells (DN; $CD3^+CD4^-CD8^-$), NK cells

(CD3$^-$CD56^{bright}CD16$^{-/dim}$ and CD3$^-$CD56^{dim}CD16$^+$), NKT cells (CD3$^+$CD56$^+$), B cells (CD3$^-$ CD56$^-$ CD19$^+$), dendritic cells (CD11b$^+$CD11c$^+$CD14$^-$), classical monocytes (CD11b$^+$CD14^{high}CD16$^-$), intermediate monocytes (CD11b$^+$CD14^{high}CD16$^+$), and non-classical monocytes (CD11b$^+$CD14^{low}CD16$^+$). (B) PBMC samples from 4 healthy donors were merged to create a t-SNE map using FlowJo software. Left panel: Immune cell subsets, defined based on the gating strategy in (A), and color-coded as indicated. Right panel: IL-10Rα expression on the immune cell subsets, with the indicated color gradient illustrating expression levels. (C) Quantification of IL-10Rα surface expression on different immune cell subsets, displayed as delta mean fluorescence intensities (ΔMFI) relative to IL-10Rα FMO controls. Data are shown as mean ± SD from 7 donors. (D) Surface expression of IL-10Rα on monocyte-derived (MoDC) immature (iDCs) and mature dendritic cells (mDCs). Data are presented as ΔMFI with mean ± SD from 4 donors. (E) Surface expression of IL-10Rα on monocyte-derived macrophage (MDM) subsets, including unpolarized (MΦ), M1-polarized, and M2-polarized macrophages, was quantified as ΔMFI. Data represent mean ± SD from 4 donors.

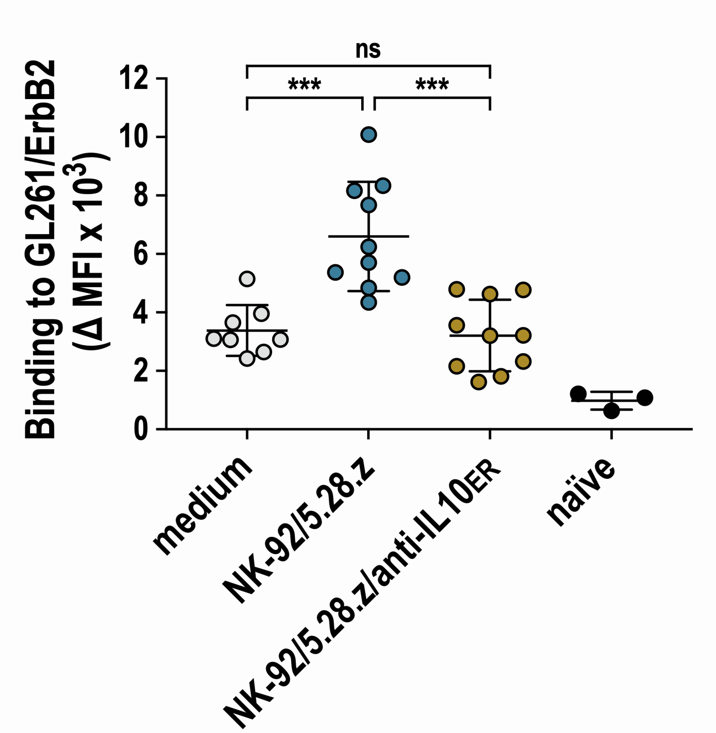

Figure S7. Tumor-specific serum antibody responses in CAR-NK-treated mice
Induction of IgG serum antibodies against GL261/ErbB2 glioblastoma cells in mice treated with injection medium (n=8), NK-92/5.28.z cells (n=10) or NK-92/5.28.z/anti-IL10ER cells (n=10) from the experiment shown in Figure 4D was investigated by flow cytometry with sera collected at the time of sacrifice, and quantified as ΔMFI relative to sera from naïve C57BL/6 mice (n=3). Results are presented as individual data points and means ± SD. ***, $p < 0.001$; ns, $p \geq 0.05$.

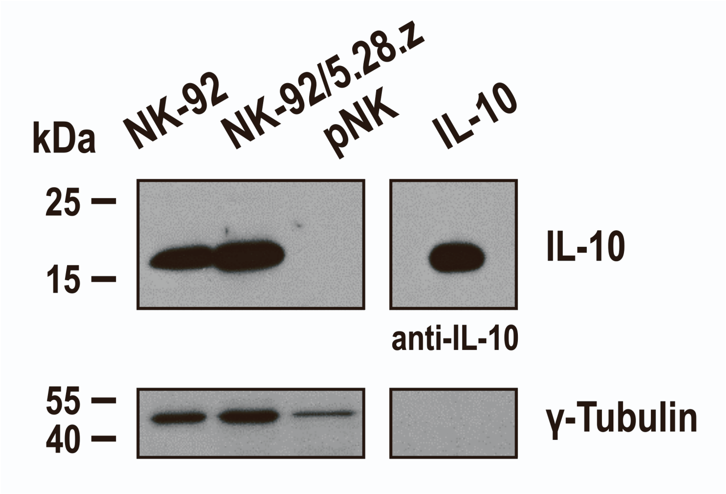

Figure S8. Activation-induced expression of IL-10 in NK cell lines and primary NK cells
Peripheral blood NK (pNK) cells from healthy donors were isolated fom buffy coats and expanded in medium containing 500 IU/mL IL-2 and 50 ng/mL IL-15 as described in Kiefer, A. et al. (2024), Cells 13, 246; doi: 10.3390/cells13030246. Parental NK-92, NK-92/5.28.z CAR-NK and *ex vivo* expanded pNK cells were activated for 5 hours with PMA/ionomycin in the presence of GolgiPlug, cell lysates were prepared and analyzed by SDS-PAGE and immunoblotting with anti-IL-10 antibody. Recombinant IL-10 was used as a positive control. γ-tubulin served as a loading control. Shown are representative data from a single pNK donor.

Supplemental Tables

Table S1. Antibodies used for multispectral imaging

Antibody	Clone	Company	Reference Number
CCR7	ERP23192-57	Abcam	ab253187
CD3ε	SP7	Abcam	ab16669
CD4	D7D2Z	Cell Signaling Technology	25229S
CD8α	D4W2Z	Cell Signaling Technology	98941
CD68	PG-M1	DAKO (Agilent Technologies)	M087601-2
CD163	EPR19518	Abcam	ab182422
CD206	polyclonal	Abcam	ab64693
FoxP3	D6O8R	Cell Signaling Technology	12653
ErbB2 (HER2)	CL0268	Atlas Antibodies	AMAb90838
IL12p35	polyclonal	Thermo Fisher Scientific	PA5-79460

Table S2. Antibodies used for flow-cytometric analysis

Antibody	Clone	Company	Reference Number
CD3	17A2	BioLegend	100220
CD3	UCHT1	BioLegend	100248
CD4	RM4-5	BD Biosciences	553051
CD4	A161A1	BioLegend	357419
CD8α	53-6.7	BioLegend	100734
CD8	SK1	BioLegend	344748
CD11b	M1/70.15.11.5	Miltenyi Biotec	130-113-236
CD11b	M1/70.15.11.5	Miltenyi Biotec	130-113-231
CD11c	B-ly6	BD Biosciences	563130
CD14	HCD14	BioLegend	325604
CD14	MϕP9	BD Biosciences	61384
CD14	MϕP9	BD Biosciences	555398
CD16	3G8	BD Biosciences	563689
CD19	HIB19	BD Biosciences	557921
CD25	3C7	BD Biosciences	558689
CD45	30-F11	BioLegend	103138
CD45	30-F11	BioLegend	103130
CD56	NCAM16.2	BD Biosciences	562751
CD56	5.1H11	BioLegend	362508
CD83	HB15e	BD Biosciences	740802
CD86	2331	BD Biosciences	555660
CD94	REA113	Miltenyi Biotec	130-123-884
CD163	GHI/61	BD Biosciences	563697
CD206	MR5D3	BD Biosciences	565250

CD210a (IL-10R)	3F9	BioLegend	308812
CD210α (IL-10R)	3F9	BD Biosciences	556013
CTLA-4	L3D10	BioLegend	349908
ErbB2 (HER2)	24D2	BioLegend	324412
F4/80	BM8	BioLegend	123110
FoxP3	MF-14	BioLegend	126419
HA-tag	16B12	BioLegend	682404
HLA-DR	G46-6	BD Biosciences	563083
IL-10	JES3-9D7	BioLegend	501404
KIR2D	NKVFS1	Miltenyi Biotec	130-123-710
LAG-3	7H2C65	BioLegend	369211
Ly6C	HK1.4	BioLegend	128032
Ly6G	1A8	BioLegend	127654
NKG2A	REA110	Miltenyi Biotec	130-113-563
NKG2C	REA205	Miltenyi Biotec	130-117-398
NKG2D	BAT221	Miltenyi Biotec	130-117-718
NKp30	REA823	Miltenyi Biotec	130-112-430
NKp44	p44-8	BD Biosciences	558564
NKp46	9 E2	Miltenyi Biotec	130-128-826
NKp80	4A4.D10	Miltenyi Biotec	130-126-102
PD-1	EH12.2H7	BioLegend	329922
PD-L1	29E.2A3	BioLegend	329718
TIGIT	MBSA43	Invitrogen	12-9500-42
TIM-3	F38-2E2	BioLegend	345026

Table S3. Antibodies used for immunofluorescence microscopy

Antibody	Clone	Company	Reference Number
HA-tag	16B12	BioLegend	682404
IL-10	JES3-9D7	BioLegend	506812
Calreticulin	EPR3924	Abcam	ab92516
CD45	2D1	Invitrogen	17-0459-42
phospho-STAT3	D3A7	Cell Signaling Technology	9145T

Table S4. Antibodies used for immunoblot analysis

Antibody	Clone	Company	Reference Number
HA-tag	C29F4	Cell Signaling Technology	11846S
γ-tubulin	polyclonal	Sigma-Aldrich	T3559
GRP78/BIP	C50B12	Cell Signaling Technology	3177S
phospho-eIF2α	D9G8	Cell Signaling Technology	3398S
eIF2α	D7D3	Cell Signaling Technology	5324S

Supplemental Methods

ImageJ Macro to quantify mean fluorescent intensity of pSTAT3 staining

Language: ImageJ Macro

```
// Set scale in microns
run("Set Scale...", "distance=84.0 known=10 unit=µm");

// Split the channels of the composite image
selectImage("1.tif");
run("Split Channels");
selectImage("C1-1.tif");
rename("Hoechst");
run("Cyan");
run("8-bit");
selectImage("C2-1.tif");
rename("Phospho-Stat3");
run("Yellow");
run("8-bit");
selectImage("C3-1.tif");
rename("CD45");
run("Magenta");
run("8-bit");

// Adjust brightness and contrast
selectImage("CD45");
run("Enhance Contrast", "saturated=0.35");
selectImage("Phospho-Stat3");
setMinAndMax(5, 117);
selectImage("Hoechst");
setMinAndMax(13, 140);

//Create a binary mask for CD45
selectImage("CD45");
setAutoThreshold("Triangle dark no-reset");
//setThreshold(7, 255);
run("Convert to Mask");
run("Close-");
run("Fill Holes");
run("Watershed");
run("Convert to Mask");

//AnalyzeParticles
run("Analyze Particles...", "size=10-Infinity circularity=0.5-1.00 display exclude clear overlay add composite");
selectImage("Phospho-Stat3");
roiManager("Measure");
```

Image-based analysis of phospho-STAT3 expression in CD45-positive cells. The macro processes multichannel fluorescence images containing Hoechst, phospho-STAT3 and CD45 channels. Images are split into individual channels, converted to 8-bit, and contrast-adjusted. The CD45 channel is thresholded and refined with morphological operations to create a mask of CD45-

positive cells. Particle analysis identifies these cells, generating ROIs, which are then applied to the phospho-STAT3 channel to quantify mean fluorescence intensity (MFI) within CD45-positive cells.
